# Chemical Composition, Antioxidant, and Antiacetylcholinesterase Activities of the Essential Oil of *Croton chimboracensis*: In Vitro and In Silico Approaches

**DOI:** 10.3390/plants15213481

**Published:** 2026-11-07

**Authors:** Claudia Cruz, Paulina Villamahua, Martín Urgilés-Recalde, Omar Malagón, Vladimir Morocho, Yunierkis Perez-Castillo

**Affiliations:** 1 Departamento de Química y Producción, Universidad Técnica Particular de Loja (UTPL), Loja 1101608, Ecuador; omalagon@utpl.edu.ec; 2 Carrera de Bioquímica y Farmacia, Universidad Técnica Particular de Loja (UTPL), Loja 1101608, Ecuador; psvillamahua@utpl.edu.ec; 3 Bio-Cheminformatics Research Group, Universidad de Las Américas, Quito 170503, Ecuador; martin.urgiles.recalde@udla.edu.ec (M.U.-R.); yunierkis.perez@udla.edu.ec (Y.P.-C.); 4 Carrera de Ingeniería Agrícola, Facultad Agropecuaria y de Recursos Naturales Renovables, Universidad Nacional de Loja, Av. Pío Jaramillo Alvarado y Av. Reinaldo Espinosa, Loja 110111, Ecuador; segundo.v.morocho@unl.edu.ec; 5 Área de Ciencias Aplicadas, Facultad de Ingeniería y Ciencias Aplicadas, Universidad de Las Américas, Quito 170503, Ecuador

**Keywords:** *Croton chimboracensis*, essential oil, sesquiterpenes, ABTS, acetylcholinesterase, molecular docking, absolute binding free energy

## Abstract

The leaf essential oil of *Croton chimboracensis*, a species native to Ecuador, was investigated using an integrated chemical, biological, and computational approach. Volatile constituents were identified by gas chromatography–mass spectrometry (GC–MS), and their relative proportions were determined by gas chromatography–flame ionization detection (GC–FID) peak-area normalization. Forty-seven compounds were identified, accounting for 95.17% of the total GC–FID peak area. The oil was dominated by hydrocarbon sesquiterpenes (72.75%), with *trans*-muurola-4(14),5-diene (34.06%), longifolene (15.34%), bicyclogermacrene (7.65%), caryophyllene oxide (5.53%), *α*-humulene (4.38%), and *γ*-cadinene (4.02%) as the principal constituents. The essential oil exhibited an assay-dependent radical-scavenging response, with an SC_50_ value of 15.20 µg/mL in the 2,2′-azinobis(3-ethylbenzothiazoline-6-sulfonic acid) (ABTS) assay (95% confidence interval CI: 8.97–19.83 µg/mL) and 2983 µg/mL in the 2,2-diphenyl-1-picrylhydrazyl (DPPH) assay (95% CI: 2719–3378 µg/mL). The essential oil also inhibited acetylcholinesterase (AChE), with an IC_50_ value of 43.78 µg/mL (95% confidence interval: 40.68–47.05 µg/mL). Molecular docking and absolute binding free-energy calculations were performed for all 47 identified constituents. These calculations prioritized spirolepechinene, *β*-phellandrene, *β*-pinene, alloaromadendrene epoxide, and (2*E*,4*E*)-nona-2,4-dien-1-ol as potential AChE-binding constituents, with predicted binding free energies of −20.22 ± 0.93, −16.59 ± 0.87, −16.33 ± 0.91, −15.81 ± 2.53, and −15.29 ± 1.91 kcal/mol, respectively (ΔG ± SEM). These findings identify candidates for subsequent experimental validation and provide molecular hypotheses concerning their possible contributions to the AChE-inhibitory activity of the complete oil. Overall, this study provides an integrated characterization of *C. chimboracensis* essential oil and establishes a basis for further investigation of its antioxidant and acetylcholinesterase-inhibitory constituents.

## 1. Introduction

The genus *Croton* L. is one of the most species-rich groups within the family Euphorbiaceae. Phylogenetic evidence supports a New World origin for the genus, followed by its diversification across tropical and subtropical regions [1]. Species of *Croton* produce essential oils (EO) with considerable interspecific variation in their volatile composition and biological properties. This diversity is evident among Ecuadorian taxa. Previous studies on *Croton* essential oils have revealed considerable variation in both chemical composition and biological activity, ranging from mixed monoterpene and sesquiterpene profiles associated with antimicrobial, radical-scavenging, and *α*-glucosidase inhibitory effects to sesquiterpene-rich oils exhibiting radical-scavenging activity and moderate acetylcholinesterase inhibition [2,3]. This variability indicates that the chemical and biological properties of an essential oil cannot be inferred solely from taxonomic affiliation and supports the individual investigation of insufficiently studied species.

*Croton chimboracensis* P.E.Berry & Riina was formally described in 2010 from western Andean Ecuador and has been documented in Chimborazo Province [4]. Despite its taxonomic recognition, the volatile composition and biological properties of its leaf essential oil have not, to the best of our knowledge, been reported in the scientific literature. Characterizing this species may therefore expand current knowledge of the phytochemical diversity of Ecuadorian *Croton*. Nevertheless, essential-oil composition may vary according to population, plant organ, developmental stage, and environmental conditions, as demonstrated in comparative studies of other *Croton* species [5]. Consequently, the profile reported in the present study should be interpreted as an initial chemical and biological baseline for the investigated plant material rather than as evidence of a species-wide chemotype.

Acetylcholinesterase (AChE) plays a central role in cholinergic neurotransmission by rapidly hydrolyzing acetylcholine and terminating synaptic signaling. Structural studies of human AChE have revealed a deep active-site gorge that accommodates pharmacologically relevant inhibitors, including donepezil [6]. Essential oils and their volatile constituents have attracted interest as sources of structurally diverse molecules capable of inhibiting this enzyme. In vitro AChE inhibition has been reported for essential oils and individual terpenes from several aromatic plants, including *Salvia lavandulaefolia* [7], different chemotypes of *C. zehntneri* [8], and *C. alnifolius* [3]. However, the inhibitory activity of a complex essential oil cannot be assigned automatically to its most abundant constituents. Comparative testing of whole oils and isolated compounds has shown that the principal components are not necessarily the strongest enzyme inhibitors [9].

The radical-scavenging properties of essential oils constitute another relevant dimension of their biological characterization. Because essential oils are chemically complex and generally contain constituents with different polarities and reaction kinetics, their responses may vary substantially among antioxidant assays. Experimental studies comparing essential oils and their constituents have demonstrated that activity measured using one radical system does not necessarily predict the response obtained with another method [9]. Therefore, the complementary use of the 2,2′-azinobis(3-ethylbenzothiazoline-6-sulfonic acid) (ABTS) and 2,2-diphenyl-1-picrylhydrazyl (DPPH) assays can provide a broader description of the in vitro radical-scavenging behavior of an essential oil, although these cell-free assays do not establish antioxidant efficacy in biological systems.

Integrating experimental enzyme inhibition with computational modeling can help investigate which constituents of a complex volatile mixture are most likely to interact with AChE. Previous studies have combined essential-oil profiling*,* in vitro AChE assays, and molecular docking to generate binding hypotheses for individual volatile constituents [10,11]. In the present study, molecular docking was followed by absolute binding free-energy calculations based on molecular dynamics simulations. This approach provides an additional energetic criterion for prioritizing plausible binders among the compounds identified in the oil [12]. Nevertheless, computational predictions should be regarded as molecular hypotheses and require subsequent validation with isolated compounds and controlled enzymatic experiments.

Accordingly, the central question of this study was whether the leaf essential oil of *C. chimboracensis* possesses a differentiated volatile profile and measurable antioxidant and AChE-inhibitory responses, and which of its identified constituents could contribute to the observed enzymatic inhibition. The aims were to characterize the volatile constituents of the oil by gas chromatography–mass spectrometry (GC–MS), determine their relative composition by gas chromatography–flame ionization detection (GC–FID) peak-area normalization, evaluate its radical-scavenging activity using the ABTS and DPPH assays, and determine its inhibitory activity against AChE. In addition, all identified constituents were evaluated by molecular docking and absolute binding free-energy calculations. We hypothesized that the observed AChE inhibition would not necessarily be explained exclusively by the most abundant components of the oil and that the computational analysis would prioritize a smaller group of potential molecular contributors for future experimental validation.

## 2. Results

### 2.1. Chemical Profile and Identification of Volatile Constituents

**Figure 1:**
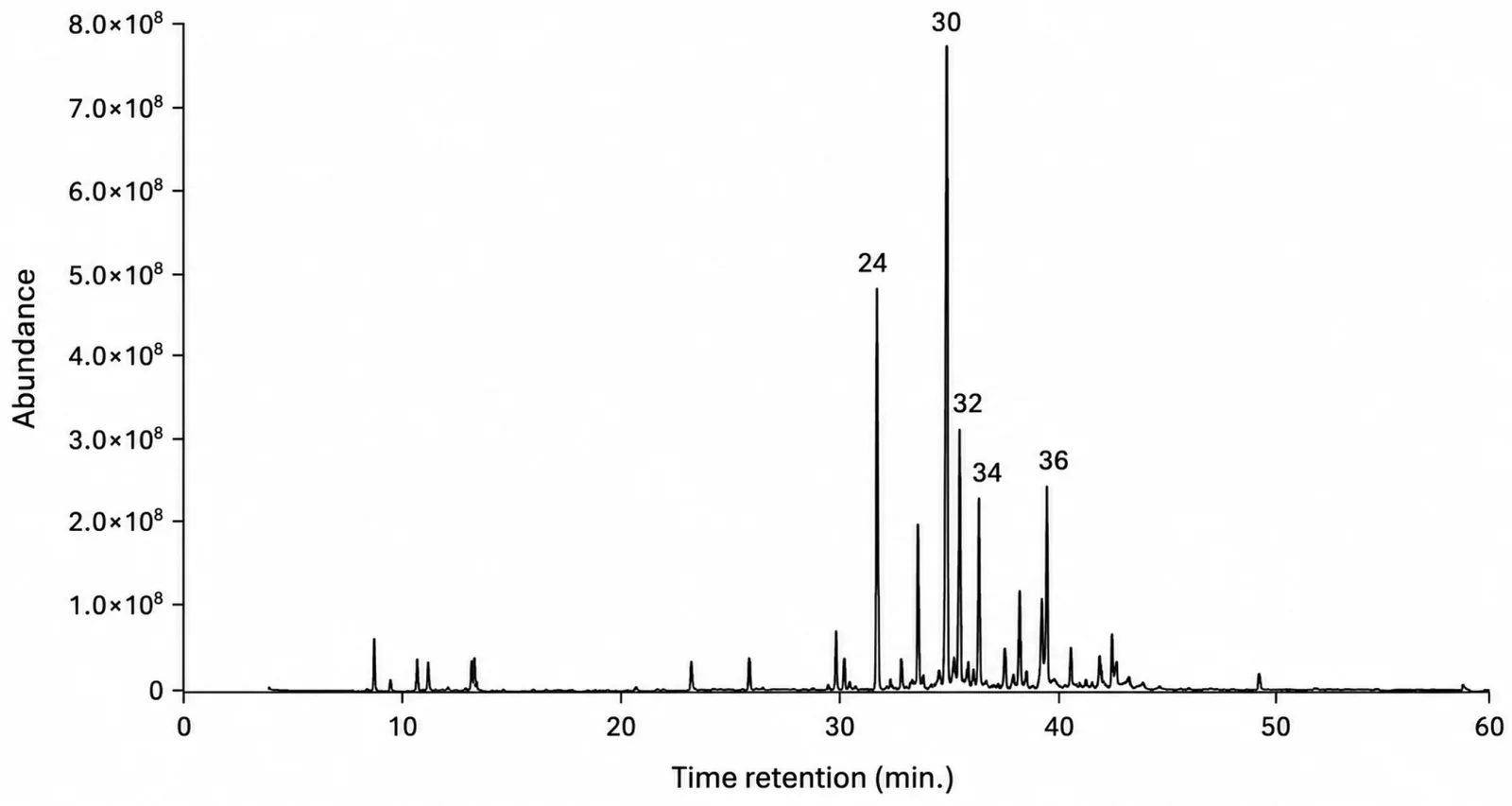
GC–MS chromatogram of the essential oil of *Croton chimboracensis* obtained using a TR-5MS capillary column. The numbers correspond to the main components listed in Table 1.

The volatile profile of the leaf essential oil of *C. chimboracensis* was investigated by GC–MS, while the relative proportions of the identified constituents were determined by GC–FID peak-area normalization. A total of 47 compounds were identified, accounting for 95.17% of the total GC–FID peak area (Figure 1). Sesquiterpene hydrocarbons were the predominant chemical class, representing 72.75% of the relative composition, followed by oxygenated sesquiterpenes (9.53%), monoterpene hydrocarbons (9.35%), other constituents (3.17%), and oxygenated monoterpenes (0.39%). Overall, sesquiterpenes accounted for 82.28% of the identified volatile fraction, demonstrating the strongly sesquiterpene-dominated character of the oil (Table 1).

The chemical structures of all 47 compounds are presented in Supplementary Figure S1, numbered according to this table.

The principal constituent was *trans*-muurola-4(14),5-diene (34.06 ± 0.83%), followed by longifolene (15.34 ± 0.40%), bicyclogermacrene (7.65 ± 0.33%), caryophyllene oxide (5.53 ± 0.45%), *α*-humulene (4.38 ± 0.21%), and *γ*-cadinene (4.02 ± 0.09%). Together, these six constituents represented 70.98% of the total GC–FID peak area, indicating that the chemical profile was largely determined by a relatively limited group of hydrocarbon and oxygenated sesquiterpenes. The main monoterpene hydrocarbons were *α*-pinene (2.16 ± 0.05%), *β*-phellandrene (1.80 ± 0.04%), *β*-pinene (1.57 ± 0.03%), myrcene (1.47 ± 0.03%), and limonene (1.26 ± 0.03%).

Compound identification was based on the combined comparison of electron-impact mass spectra with the NIST 17 library and literature spectra, together with experimentally calculated linear retention indices and the reference values reported by Adams [13]. Absolute differences between experimental and reference indices ranged from 1 to 19 units across the 47 assigned constituents. For the five computationally prioritized compounds, these differences were 1 unit for spirolepechinene, 9 for *β*-phellandrene, 5 for *β*-pinene, 7 for alloaromadendrene epoxide, and 16 for (2*E*,4*E*)-nona-2,4-dien-1-ol. Retention-index agreement was considered supporting evidence alongside mass-spectral matching, rather than an independent confirmation of identity. Assignments with larger discrepancies require greater caution. Furthermore, analysis on a single TR-5MS column cannot completely exclude co-elution or ambiguity among structurally related constituents. Accordingly, the computational predictions should be interpreted as conditional on the assigned molecular identities.

The predominance of sesquiterpenes is consistent with the composition reported for several essential oils from the genus *Croton*, although substantial differences occur in the identity and abundance of their major constituents [14]. For example, the essential oil of the Ecuadorian species *C. ferrugineus* was characterized mainly by (*E*)-caryophyllene, myrcene, *β*-phellandrene, germacrene D, and *α*-humulene [2]. The oil of *C. alnifolius* also contained a high proportion of sesquiterpene hydrocarbons, but its principal compounds were (*E*)-caryophyllene, *α*-pinene, germacrene D, myrcene, and *β*-chamigrene [3]. Similarly, the sesquiterpenoid-rich oil of *C. hirtus* was dominated by *β*-caryophyllene, germacrene D, *β*-elemene, *α*-humulene, and caryophyllene oxide [14].

Therefore, although sesquiterpene accumulation is a recurrent feature among several *Croton* essential oils, the predominance of *trans*-muurola-4(14),5-diene and longifolene differentiates the chemical profile of *C. chimboracensis* from those previously reported for other species of the genus. These findings expand current knowledge of the chemical diversity of *Croton* essential oils and provide the first characterization of the volatile profile of *C. chimboracensis.*

### 2.2. Antioxidant Activity

The radical-scavenging activity of the leaf essential oil of *C. chimboracensis* was evaluated using the ABTS and DPPH assays. In the ABTS assay, the essential oil exhibited an SC_50_ value of 15.20 µg/mL (95% CI: 8.97–19.83 µg/mL; Hill slope = 1.345; R^2^ = 0.9820). The antioxidant capacity measured using this assay was also expressed as a Trolox equivalent antioxidant capacity (TEAC) of 21.53 ± 1.79 µmol Trolox equivalents/g essential oil. In contrast, the SC_50_ value obtained using the DPPH assay was 2983 µg/mL (95% CI: 2719–3378 µg/mL; Hill slope = 1.855; R^2^ = 0.9971). Thus, the essential oil required a substantially lower concentration to reduce the ABTS radical cation by 50% than to achieve the same radical-scavenging response in the DPPH assay. This difference indicates that the radical-scavenging activity of the essential oil was strongly assay-dependent. The experimental values and the corresponding Trolox controls are presented in Table 2.

**Figure 2:**
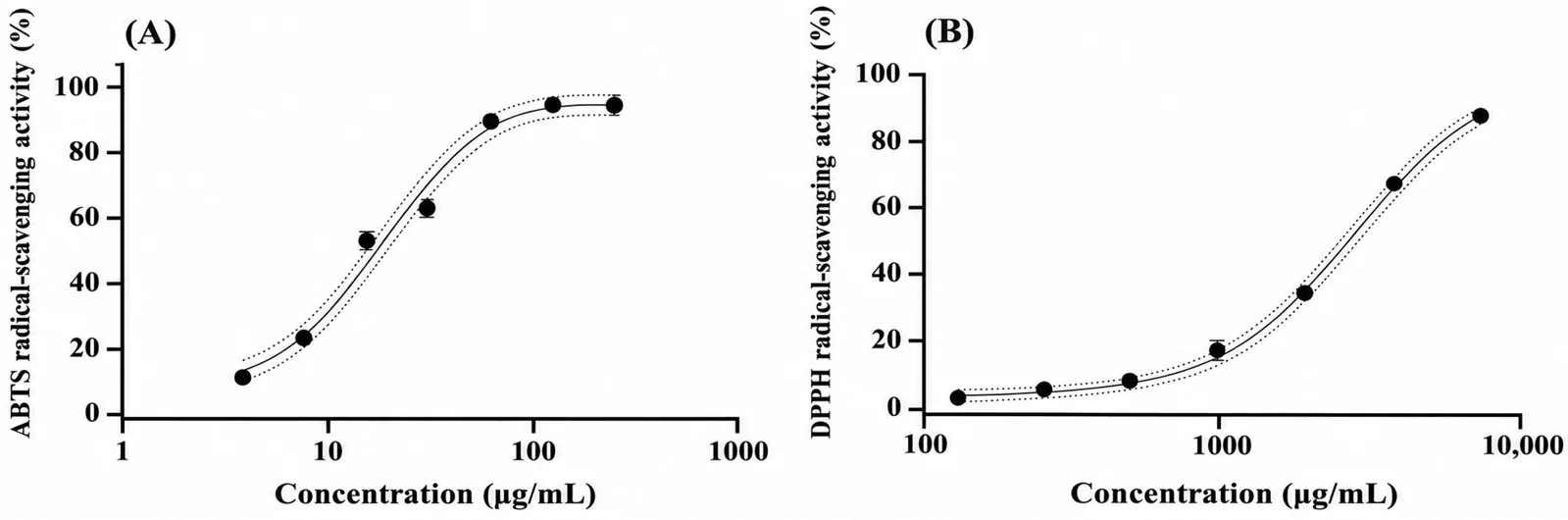
Concentration–response curves for the radical-scavenging activity of the leaf essential oil of *C. chimboracensis* in the (**A**) ABTS and (**B**) DPPH assays. The solid line represents the fitted nonlinear regression curve, and the dashed lines represent the 95% confidence interval.

The experimental data were fitted by nonlinear regression using a four-parameter logistic model with variable slope to estimate the SC_50_ values and their 95% confidence intervals (Figure 2). Individual replicate values (n = 3), together with the mean, standard deviation (SD), and standard error of the mean (SEM) for each concentration, are provided in Supplementary Table S1.

The substantially different SC_50_ values obtained in the ABTS and DPPH assays describe an assay-dependent response under the experimental conditions employed. They should not be interpreted as evidence of a specific antioxidant mechanism or as directly interchangeable measures of antioxidant potency. Differences in radical chemistry, reaction kinetics, and sample behavior in the reaction media may contribute to this contrast [9]. Sample dispersion and potential optical interference also require consideration, particularly at the higher essential-oil concentrations used in the DPPH assay. Consequently, the observed difference should not be attributed exclusively to radical reactivity without accounting for these analytical factors.

The ABTS SC_50_ obtained for *C. chimboracensis* was numerically lower than those reported for several other *Croton* essential oils. For example, *C. alnifolius* showed an ABTS SC_50_ of 28.43 ± 1.00 µg/mL [3], while values of 901 ± 20 µg/mL and 453.85 ± 15.87 µg/mL were reported for *C. ferrugineus* [2] and *C. thorelii* [15], respectively. The essential oil of *C. conduplicatus*, collected during both dry and rainy seasons, also exhibited a more pronounced response in the ABTS assay than in DPPH and *β*-carotene/linoleic acid assays, although its results were reported as percentage inhibition and are therefore not directly comparable with the SC_50_ value obtained here [16].

In the DPPH assay, the response of *C. chimboracensis* was considerably weaker. Its SC_50_ value was higher than that reported for *C. alnifolius* (1903.29 ± 1.26 µg/mL) [3], but lower than the value reported for *C. piauhiensis* (4623.83 µg/mL) [17]. The latter oil displayed different responses across antioxidant models, with an EC_50_ of 171.21 µg/mL in the *β*-carotene bleaching assay, illustrating that an oil showing limited DPPH scavenging may respond differently in a lipid-peroxidation model [17]. In contrast, essential oils from *C. argyrophylloides*, *C. jacobinensis*, *C. nepetifolius*, and *C. sincorensis* exhibited substantially lower DPPH IC_50_ values, ranging from 12.55 to 80.59 µg/mL [18].

The considerable variability among these studies reinforces the need for caution when comparing antioxidant values from different publications. Differences in radical concentration, solvent composition, incubation time, sample concentration range, calculation method, and positive control may substantially affect the reported endpoint. Moreover, studies on *C. matourensis* demonstrated antioxidant responses in DPPH assays using hydrodistilled, hexane, and supercritical CO_2_ extracts, with the latter containing nonvolatile compounds and phenolic constituents not necessarily present in an essential oil [19]. Similarly, free and solid-lipid-nanoparticle-loaded essential oil of *C. argyrophyllus* showed DPPH scavenging and inhibition of lipid peroxidation, but those results were influenced by the formulation and cannot be directly compared with the unformulated oil evaluated here [20].

Although the essential oil of *C. chimboracensis* was dominated by hydrocarbon sesquiterpenes, its ABTS radical-scavenging activity cannot be attributed to *trans*-muurola-4(14),5-diene, longifolene, bicyclogermacrene, caryophyllene oxide, or any other individual constituent because the antioxidant assays were performed only with the whole oil. Previous studies comparing essential oils with their isolated major constituents have shown that the activity of the complete mixture may differ from that of its individual components and that the relationship between volatile composition and antioxidant response is not always straightforward [9,21]. Moreover, interactions among major, minor, and trace constituents may influence the final biological response of essential oils [13]. Therefore, the present results do not allow the contribution of individual compounds to be established or the interactions among them to be classified as additive, synergistic, or antagonistic.

Overall, the essential oil of *C. chimboracensis* exhibited a marked ABTS•^+^ scavenging response and substantially weaker activity against DPPH•. This contrast confirms that its measured antioxidant behavior was dependent on the radical system and experimental conditions employed. Within the scope of the present study, these findings complement the chemical characterization and acetylcholinesterase inhibition results, whereas the computational analysis was specifically directed toward identifying plausible molecular contributors to the observed enzymatic activity.

### 2.3. Acetylcholinesterase Inhibitory Activity

The acetylcholinesterase (AChE) inhibitory activity of the leaf essential oil of *Croton chimboracensis* was evaluated using a modified Ellman-based colorimetric assay with kinetic monitoring at 412 nm. Enzymatic reaction rates were determined from the slopes of absorbance–time plots (ΔA/min) during the initial 10 min reaction interval, and the corresponding inhibition percentages were calculated relative to the solvent-matched enzyme control.

**Figure 3:**
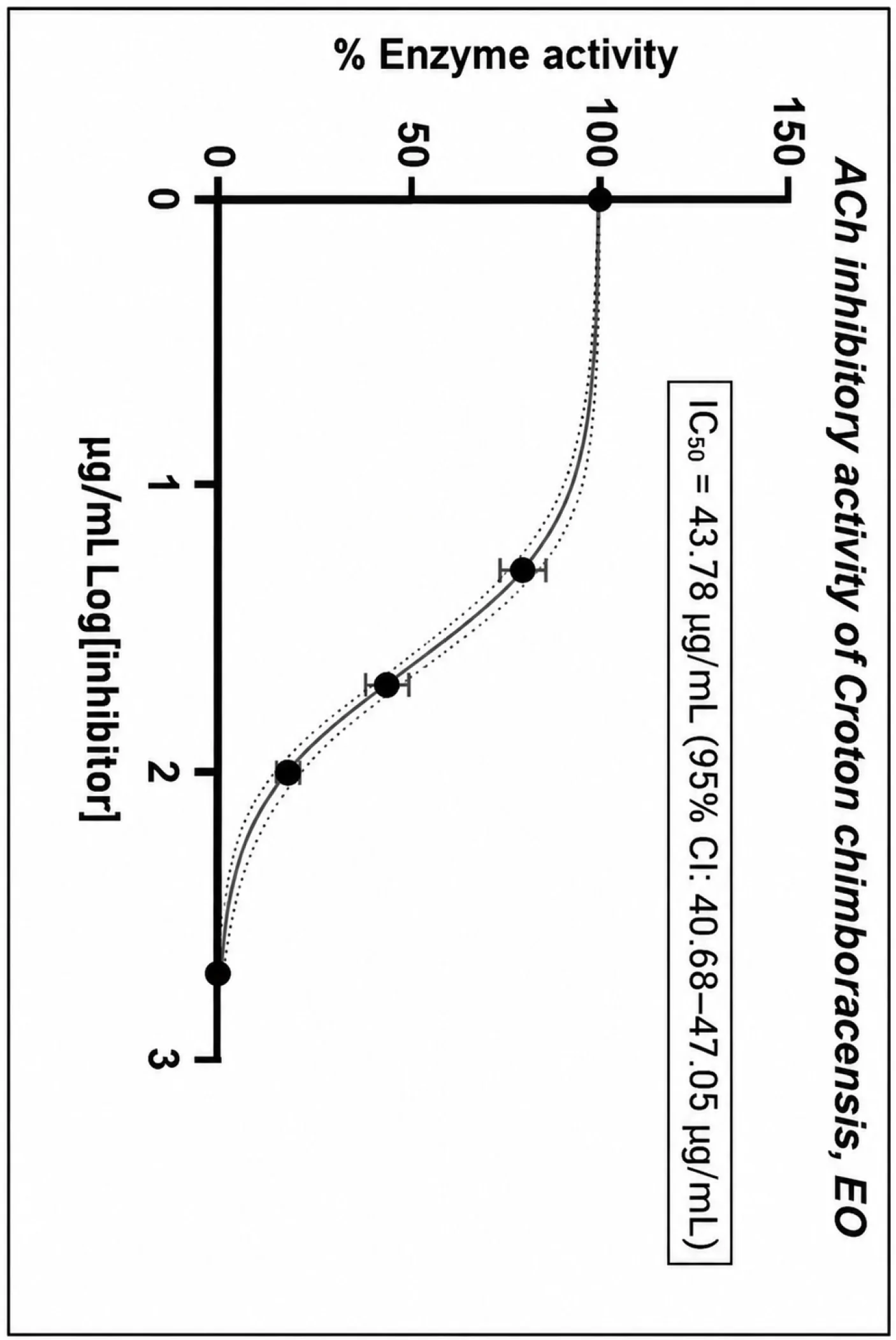
Concentration–response curve for acetylcholinesterase (AChE) inhibition by the leaf essential oil of *Croton chimboracensis*. Inhibition percentages were calculated from enzymatic reaction rates determined by linear regression of absorbance versus time during the initial 10 min interval. The solid line represents the fitted nonlinear regression curve, and the dashed lines represent the 95% confidence interval.

The concentration–response relationship was evaluated at five final essential oil concentrations (1, 20, 50, 100, and 500 µg/mL). Nonlinear regression analysis was performed using a four-parameter logistic (4PL) model with variable slope in GraphPad Prism version 11.1.0. The fitted concentration–response curve yielded an IC_50_ value of 43.78 µg/mL (95% confidence interval: 40.68–47.05 µg/mL), indicating concentration-dependent inhibition of AChE under the experimental conditions employed (Figure 3).

The IC_50_ was estimated using nonlinear regression. Essential oil concentrations are expressed in µg/mL. The IC_50_ obtained for *C. chimboracensis* was numerically lower than that reported for the essential oil of *C. alnifolius* (61.74 ± 1.02 µg/mL) [3] and comparable to the values reported by Hung et al. for several essential oils, with the most active samples exhibiting IC_50_ values ranging from 28.71 to 58.38 µg/mL [22]. According to the classification proposed by Magalhães et al. (2021), the inhibitory activity of *C. chimboracensis* can be considered moderate [23]. Acetylcholinesterase is a key enzyme in cholinergic neurotransmission, responsible for the rapid hydrolysis of acetylcholine at the synaptic cleft [24]. Its inhibition increases acetylcholine availability and represents an established pharmacological strategy for the symptomatic management of Alzheimer’s disease. Accordingly, AChE inhibitors such as donepezil, rivastigmine, and galantamine remain clinically relevant treatments [25]. Essential oils and their volatile constituents have also attracted interest because several monoterpenes and sesquiterpenes exhibit cholinesterase-inhibitory activity and may contribute to broader neuroprotective effects [26,27]. In this context, the activity observed for *C. chimboracensis* suggests the presence of bioactive constituents of pharmacological interest.

Nevertheless, comparisons between studies should be interpreted cautiously, as IC_50_ values may be influenced by the enzyme source and concentration, substrate concentration, incubation time, solvent system, and concentration–response analysis method [22,28].

Studies of AChE inhibition within the genus *Croton* remain limited and have employed different sample types and analytical methods. Essential oils obtained from the leaves, stems, and roots of three *C. zehntneri* chemotypes exhibited AChE inhibition in a thin-layer chromatography (TLC) bioautographic assay [8]. However, because this method provides qualitative or semiquantitative results, direct comparison with the IC_50_ determined for *C. chimboracensis* is not appropriate. Compounds isolated from the ethyl acetate fraction of *C. sylvaticus* showed IC_50_ values ranging from 60.7 to 415.0 µg/mL, whereas its crude extract and solvent fractions exhibited values between 235.0 and 4695.0 µg/mL [29].

AChE inhibition has also been reported for leaf extracts and solvent fractions of *C. gratissimus* and *C. zambesicus*, with stronger inhibitory activity generally observed for the former [30]. These findings indicate that chemically diverse preparations from several *Croton* species contain AChE-inhibitory constituents. However, comparisons with the present study remain contextual, as solvent extracts contain nonvolatile compounds, particularly flavonoids and other phenolic constituents, which differ chemically from the terpenoid-rich essential oil of *C. chimboracensis*. Moreover, differences in experimental conditions, including the solvent system and sample dispersion, may also influence the inhibitory responses reported across studies.

In the present study, although the essential oil reaction mixtures appeared visually homogeneous, this qualitative observation does not confirm complete solubilization or dispersion stability in the aqueous medium. Furthermore, while a solvent-matched control was included, the effect of 10% (*v/v*) methanol on intrinsic AChE activity was not independently evaluated. Therefore, potential influences associated with solvent conditions and the physicochemical behavior of the essential oil cannot be excluded.

Overall, the leaf essential oil of *C. chimboracensis* exhibited moderate in vitro AChE inhibitory activity, with the IC_50_ reflecting the response of the complete volatile mixture under the experimental conditions employed, without attributing this activity to individual constituents. Further studies using optimized solvent conditions and independently validated kinetic assays are warranted to confirm its inhibitory potential.

The absorbance–time profiles recorded at 412 nm during the initial 10 min reaction interval are provided in Supplementary Figure S2. Linear regression parameters, including reaction-rate slopes (ΔA/min), intercepts, and coefficients of determination (R^2^) for individual replicates, are presented in Supplementary Table S2. The corresponding nonlinear concentration–response regression parameters, including the estimated IC_50_, 95% confidence interval, Hill slope, and lack-of-fit test results, are provided in Supplementary Table S3.

### 2.4. Molecular Modeling

To investigate which constituents of *C. chimboracensis* essential oil could contribute to the observed inhibitory activity against acetylcholinesterase from *Electrophorus electricus* (*ee*AChE), a sequential computational strategy was applied. The 47 compounds identified in the essential oil were first docked to *ee*AChE to generate binding hypotheses. These were later evaluated by Absolute Binding Free Energy (ABFE) calculations based on alchemical molecular dynamics (MD) simulations. The ABFE values were the criterion employed to prioritize the molecules with the best potential of binding to eeAChE independently from their relative abundance.

Molecular docking is particularly useful in virtual screening settings, where a large set of compounds must be rapidly prioritized against a single receptor. In this context, docking provides a computationally efficient strategy to generate plausible binding poses and identify candidates with favorable geometric and chemical complementarity within the binding cavity [31]. However, its scoring functions provide very approximate estimates of binding affinity because they do not fully account for solvent effects, conformational flexibility, entropic contributions, or the energetic cost associated with ligand and receptor reorganization. In contrast, ABFE methods are among the most rigorous computational approaches for estimating protein–ligand binding because they use alchemical molecular dynamics simulations to quantify the free energy difference between the protein-bound and solvated unbound states. Although computationally more demanding, ABFE calculations can provide higher accuracy than docking methods [32].

Molecular docking calculations were carried out for all 47 compounds against the eeAChE model to generate binding hypotheses. In total, 96 docking complexes were selected for further analyses according to the approach described in the Materials and Methods section. The simplified molecular input line entry system (SMILES) codes for each compound and the docking results for all complexes are provided in Supplementary Materials Tables S4 and S5, respectively.

**Figure 4:**
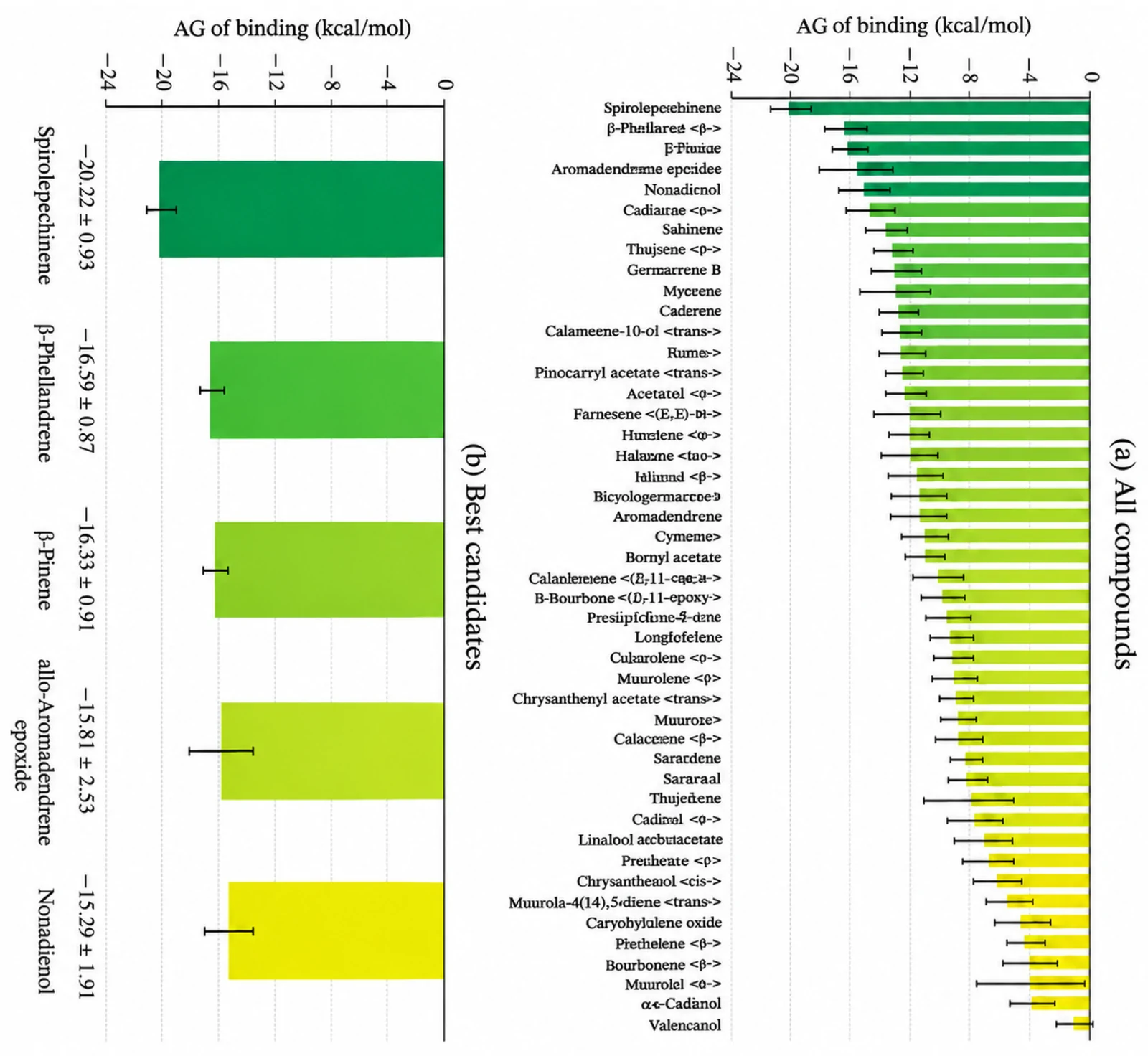
Results of ABFE calculations for eeAChE. (**a**) The most negative ABFE estimate obtained among the evaluated docking-selected poses of each of the 47 identified constituents. (**b**) The five constituents prioritized according to this criterion. Values are presented as ΔG ± SEM in kcal/mol. The abbreviated label “Nonadienol” denotes (2*E*,4*E*)-nona-2,4-dien-1-ol.

The results of the ABFE calculations are summarized in Figure 4a for the most negative binding free energy estimate of each compound, while full results for all 96 docking-predicted complexes are given in Table S6 of the Supplementary Materials. The five compounds with the most negative binding energy estimates were spirolepechinene, *β*-phellandrene, *β*-pinene, alloaromadendrene epoxide, and nonadienol (Figure 4b). These were prioritized as potential eeAChE-binding constituents among the compounds present in the essential oil.

ABFE estimates were sensitive to the docking-selected starting poses. The differences between the most favorable and the next-most favorable evaluated pose were 7.63 kcal/mol for spirolepechinene, 5.35 kcal/mol for *β*-phellandrene, 14.37 kcal/mol for *β*-pinene, and 3.24 kcal/mol for (2*E*,4*E*)-nona-2,4-dien-1-ol. Only one pose was retained for alloaromadendrene epoxide, preventing an assessment of its sensitivity to alternative starting poses. Consequently, the reported ranking is conditional on the evaluated poses and should not be interpreted as a pose-independent affinity ranking. The block-based uncertainties reported by BAT.py characterize numerical variability within each calculation; they do not encompass uncertainty associated with alternative poses, receptor-model accuracy, force-field approximations, or incomplete sampling.

Donepezil was used as a benchmark ligand based on its reported inhibitory activity against human acetylcholinesterase (*hs*AChE) and eeAChE*,* and on the availability of its crystallographic binding mode to hsAChE (Protein Data Bank (PDB) ID 4EY7) [6,33]. For this aim, the pose of donepezil in the complex with hsAChE was used as a reference for constrained docking to the *E. electricus* enzyme. The constraints for shape overlap and for hydrogen bond acceptors and donors were set to 100. ABFE was then evaluated for the resulting donepezil–eeAChE complex, yielding −17.03 ± 2.40 kcal/mol. 

The docking protocol was assessed by unconstrained redocking of donepezil into hsAChE using the settings described in the Methods section. The top-ranked pose was the only solution with an AggScore > 1 and had a ligand heavy-atom RMSD of 1.98 Å relative to the crystallographic pose in 4EY7, supporting recovery of a near-crystallographic binding mode. ABFE calculations were performed using the crystallographic pose and docking poses ranked second, third, and fourth as starting structures. These three docked poses were included as additional controls to assess sensitivity of ABFE calculations to the initial ligand configuration, although they did not meet the AggScore selection threshold. The resulting ABFE estimates were −18.50 ± 3.20 kcal/mol for the crystallographic pose, and −9.04 ± 2.02, −5.35 ± 3.45, and −5.94 ± 3.28 kcal/mol for docking poses 2, 3, and 4, respectively. The crystallographic starting structure yielded a more favorable estimate than the three alternative poses. These results support structural pose recovery and provide a computational comparison of alternative binding configurations, but do not establish quantitative agreement with experimental binding affinities.

These five computationally prioritized compounds represent chemically distinct constituents of the essential oil: spirolepechinene is a hydrocarbon sesquiterpene, *β*-phellandrene and *β*-pinene are hydrocarbon monoterpenes, alloaromadendrene epoxide is an oxygenated sesquiterpene, and (2*E*,4*E*)-nona-2,4-dien-1-ol is an oxygenated aliphatic constituent. Their relative GC–FID peak areas were 0.35%, 1.80%, 1.57%, 0.15%, and 0.13%, respectively, accounting collectively for 4.00% of the total peak area. This ranking identifies candidates for experimental validation but does not establish their contributions to the inhibitory activity of the complete oil. Such contributions depend on the concentration available in the assay, experimentally determined inhibitory potencies, and possible interactions within the mixture.

These compounds have been previously reported as constituents of essential oils associated with antioxidant [34], anti-inflammatory [35], antimicrobial [36] and AChE inhibitory activities [37]. Among them, *β*-phellandrene and *β*-pinene have been experimentally confirmed as *ee*AChE inhibitors*,* with reported IC_50_ values of 0.882 mM [38] and 1.1 mM [37], respectively. These literature observations provide biological context but do not validate the present ABFE predictions. No isolated-compound AChE measurements were performed in the present study. Thus, spirolepechinene, alloaromadendrene epoxide, and (2*E*,4*E*)-nona-2,4-dien-1-ol remain computationally prioritized AChE inhibitor candidates.

To place these relative abundances in the context of the enzymatic assay, approximate nominal constituent concentrations can be calculated by multiplying the final essential-oil concentration by the corresponding relative GC–FID peak-area fraction. This calculation assumes that peak-area fractions approximate mass fractions and is therefore illustrative rather than a calibrated quantification. At the reported whole-oil IC_50_ of 43.78 µg/mL, the estimated nominal concentrations would be approximately 0.059 µg/mL for spirolepechinene, 0.305 µg/mL for *β*-phellandrene, 0.266 µg/mL for *β*-pinene, 0.025 µg/mL for alloaromadendrene epoxide, and 0.022 µg/mL for (2*E*,4*E*)-nona-2,4-dien-1-ol. These values are neither individual-compound IC_50_ values nor measurements of freely dissolved concentrations in the assay medium. Accordingly, the computational ranking cannot establish whether these constituents inhibit AChE at the concentrations potentially present in the tested oil. Their individual concentration–response relationships require experimental evaluation.

**Figure 5:**
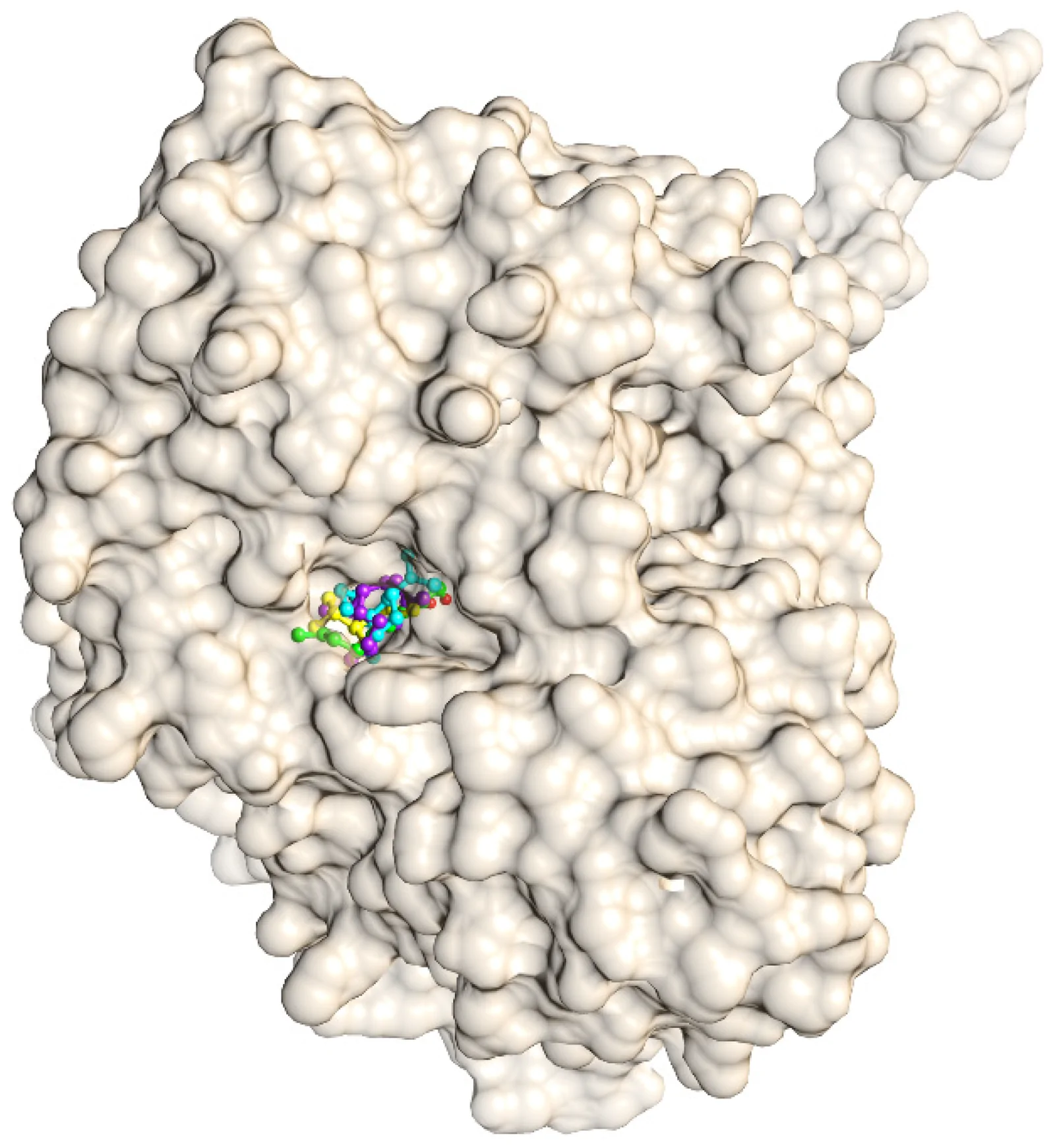
Predicted binding poses of the prioritized compounds to eeAChE. The superposition of all compounds in the binding cavity is shown as multicolor sticks: *β*-pinene-orange, *β*-phellandrene-yellow, (2*E*,4*E*)-nona-2,4-dien-1-ol-green, spirolepechinene-cyan, alloaromadendrene epoxide-purple.

Considering these results, the predicted binding modes of the five prioritized compounds to *ee*AChE were analyzed in detail. The representative structure of each complex was defined as the centroid of the most populated cluster obtained after clustering the MD snapshots of the last equilibration step performed by BAT.py before proceeding to the alchemical calculations. This analysis shows that all five compounds occupy the same binding cavity and share a predominantly hydrophobic interaction pattern with *ee*AChE, as shown in Figure 5.

**Figure 6:**
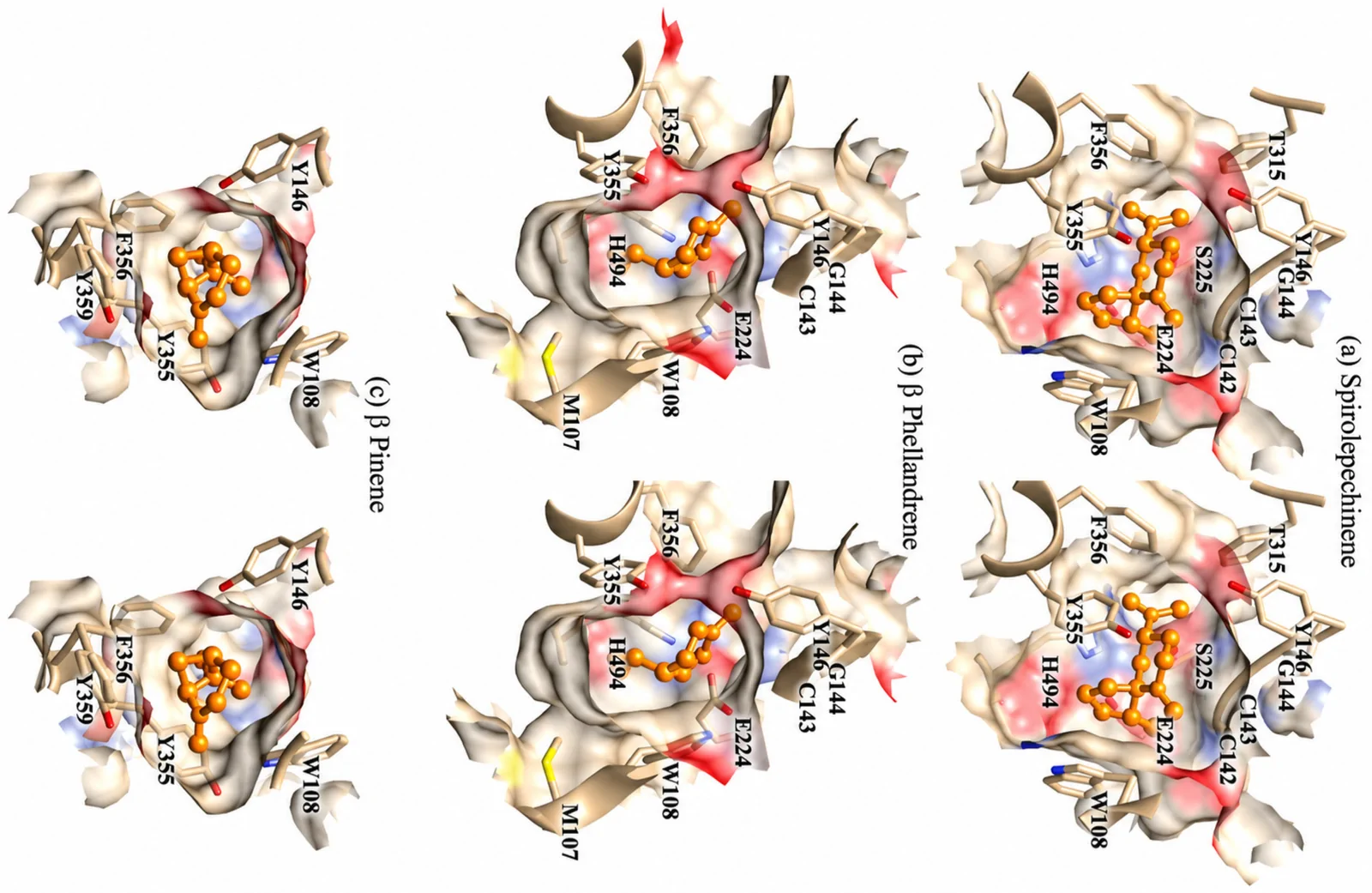
Cross-eye stereo representation of the predicted binding poses and ligand-receptor interactions for the fully hydrocarbon-prioritized compounds, represented as orange balls and sticks: (**a**) spirolepechinene, (**b**) *β*-phellandrene and (**c**) *β*-pinene. Only residues interacting with the compound in at least 50% of the analyzed MD snapshots are labeled.

**Figure 7:**
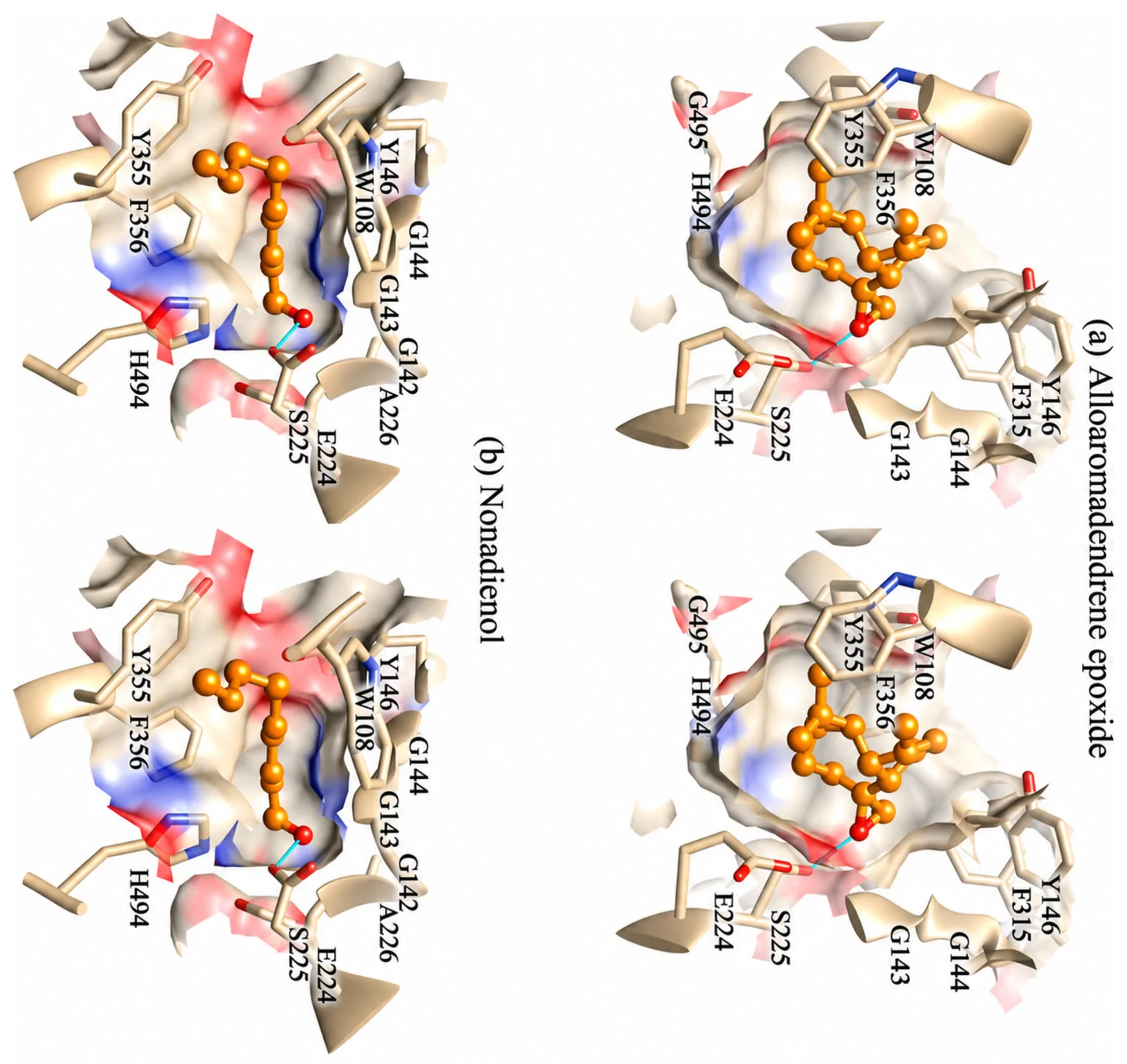
Cross-eye stereo representation of the predicted binding poses and ligand-receptor interactions for the oxygen-containing ligand, represented as orange balls and sticks: (**a**) alloaromadendrene epoxide and (**b**) (2*E*,4*E*)-nona-2,4-dien-1-ol. Only residues interacting with the compound in at least 50% of the analyzed MD snapshots are labeled. Hydrogen bonds are represented as cyan lines.

Overall, all five compounds are predicted to interact with W108, Y355 and F356 through van der Waals and π–alkyl contacts in the case of carbon-rich ligand scaffolds. Additional common interacting residues include F315, Y146, and H494. For the fully hydrocarbon ligands spirolepechinene, *β*-phellandrene and *β*-pinene, their binding modes were driven by hydrophobic contacts, as shown in Figure 6. In contrast, the oxygen-containing ligands alloaromadendrene epoxide and (2*E*,4*E*)-nona-2,4-dien-1-ol introduced polar interactions, and both ligands contact the backbone atoms of G143, G144, and H494, as presented in Figure 7.

As shown in Figure 6, in the *ee*AChE-Spirolepechinene predicted complex, the compound interacts with E224, G142, G143, G144 too. A similar interaction pattern was observed for *β*-phellandrene, where one aliphatic edge of the monoterpene scaffold is located close to E224 and H494. The hydrocarbon core for this compound is packed against G143, G144 and F356. The other fully hydrocarbon ligand, *β*-pinene, interacts with Y359 and F356, in addition to the residues interacting with all analyzed compounds. Finally, in the predicted complexes for the oxygenated compounds (Figure 7), the epoxide oxygen of alloaromadendrene epoxide was positioned close to S225, forming a hydrogen bond with its side chain and interacting with G495, whereas (2*E*,4*E*)-nona-2,4-dien-1-ol terminal oxygen forms a hydrogen bond with the side chain of E224 and interacts with peripheral residues A226 and G142.

Previous research has shown that donepezil inhibits both *ee*AChE and hsAChE [6,36]. Furthermore, the binding sites in both enzymes are highly conserved. To explore the predicted binding of the five prioritized constituents to the human enzyme, they were evaluated against hsAChE. The same modeling protocol employed for the *ee*AChE enzyme led to binding free energies to hsAChE close to that predicted for the hsAChE-donepezil benchmark complex (−18.5 kcal/mol). The obtained values were: (2*E*,4*E*)-nona-2,4-dien-1-ol −18.58 kcal/mol, spirolepechinene −17.52 kcal/mol, alloaromadendrene epoxide −17.40 kcal/mol, *β*-pinene −13.37 kcal/mol and *β*-phellandrene −12.15 kcal/mol. Among these compounds, previous studies reported 68% inhibition of hsAChE by *β*-pinene at 4.7 mM [7]. These results suggest that constituents of the *C. chimboracensis* essential oil prioritized for *ee*AChE inhibition could also potentially bind hsAChE. The five constituents require experimental evaluation against hsAChE under matched assay conditions

AChE is a key enzyme in cholinergic neurotransmission because it rapidly hydrolyzes acetylcholine into choline and acetate, thereby terminating synaptic signaling [39,40]. Inhibition of AChE reduces acetylcholine hydrolysis and increases its availability at cholinergic synapses, which explains the pharmacological relevance of AChE inhibitors such as donepezil in the symptomatic treatment of Alzheimer’s disease [41]. The modeling results suggest that the *ee*AChE inhibitory activity observed for the essential oil of *C. chimboracensis* could be related to spirolepechinene, alloaromadendrene epoxide and (2*E*,4*E*)-nona-2,4-dien-1-ol, in addition to the previously confirmed AChE inhibitors *β*-phellandrene and *β*-pinene. Moreover, their favorable predicted profiles against hsAChE expand the scope of these compounds as candidates with potential relevance for the human enzyme.

## 3. Materials and Methods

### 3.1. Plant Material

Leaves of *C. chimboracensis* P.E.Berry & Riina were collected in October 2024, during the flowering stage, in Susudel, Oña Canton, Azuay Province, Ecuador, at 3.4054305° S, 79.1837746° W and approximately 2319 m above sea level. The plant material was transported to the Department of Chemistry of the Universidad Técnica Particular de Loja, Loja, Ecuador. Collection was conducted under authorization No. MAATE-ARSFC-2022-2839, issued by the Ministry of Environment and Energy of Ecuador. Taxonomic identification was confirmed by Dr. Nixon Cumbicus at the Herbarium of the Universidad Técnica Particular de Loja. A voucher specimen was deposited in the same herbarium under accession number HUTPL5626. The leaves were separated from the remaining plant material and dried in an electric oven (Lassele DY-330H, Ansan City, Gyeonggi-do, Republic of Korea) at 35 °C for 48 h. The dried plant material was immediately used for essential oil extraction.

### 3.2. Extraction of Essential Oil

The essential oil was obtained from dried leaves of *C. chimboracensis* by steam distillation for 4 h using a vertical distillation apparatus. Three independent distillations were performed, each using 200 g of dried plant material. After each distillation, the essential oil was separated from the aqueous phase, transferred to an amber glass vial, and stored at approximately −4 °C. The chemical and biological analyses were performed immediately after essential oil extraction. The extraction yield was calculated for each independent distillation on a weight-to-dry-weight basis and expressed as mean ± standard deviation (% *w/w*).

### 3.3. Chemical Characterization of the Essential Oil

#### 3.3.1. Gas Chromatography–Mass Spectrometry (GC–MS) Analysis

For qualitative chemical profiling, 10 µL of the essential oil was dissolved in 990 µL of HPLC-grade cyclohexane to obtain a 1% (*v*/*v*) solution (Sigma-Aldrich, St. Louis, MO, USA). The analysis was performed using a Thermo Scientific Trace 1310 gas chromatograph coupled to an ISQ 7000 single-quadrupole mass spectrometer (Thermo Fisher Scientific, Waltham, MA, USA). Data acquisition and processing were carried out using the Chromeleon chromatography data system. Chromatographic separation was achieved using a non-polar TR-5MS capillary column (5% phenyl polysilphenylene-siloxane stationary phase; 30 m × 0.25 mm internal diameter × 0.25 µm film thickness; Thermo Fisher Scientific, Waltham, MA, USA). Helium was used as the carrier gas at a constant flow rate of 1.0 mL/min (Indura, Guayaquil, Ecuador). A 1 µL aliquot of the diluted essential oil was injected in split mode at a split ratio of 1:80, and the injector temperature was maintained at 230 °C. The oven temperature was initially set at 50 °C and held for 3 min, increased to 230 °C at a rate of 3 °C/min, and maintained at the final temperature for 3 min. The mass spectrometer was operated in electron-impact ionization mode at 70 eV and in full-scan mode over an *m*/*z* range of 40–400. The ion-source and transfer-line temperatures were maintained at 230 and 250 °C, respectively.

The volatile constituents were identified by combined comparison of their electron-impact mass spectra with those contained in the NIST 17 mass spectral library and of their experimentally calculated linear retention indices with published values, primarily those compiled by Adams [13]. Linear retention indices were calculated according to the Van den Dool and Kratz equation [42], based on the retention times of a homologous series of n-alkanes C9–C22 (TPH-6RPM; Chem Service, West Chester, PA, USA) analyzed under the same chromatographic conditions as the essential oil.

Data acquisition and processing were performed using Chromeleon XPS software, version 7.2.10 (Thermo Fisher Scientific, Waltham, MA, USA), and mass-spectral matching was carried out using the NIST 17 MS library integrated into the chromatographic data system. The combined use of mass-spectral and retention-index data followed the analytical approach previously applied to volatile compounds and essential oils [43,44].

#### 3.3.2. Gas Chromatography–Flame Ionization Detection Analysis

The relative composition of the essential oil was determined using a Thermo Scientific Trace 1310 gas chromatograph equipped with a flame ionization detector. The TR-5MS capillary column, essential-oil dilution, injection conditions, carrier-gas flow rate, injector temperature, and oven temperature program were identical to those described for the GC–MS analysis. The essential oils obtained from the three independent distillations were analyzed separately. The relative proportion of each constituent was calculated by normalization of the corresponding integrated GC–FID peak area. The results were expressed as mean relative peak area (%) ± standard deviation from the three independent extraction replicates (n = 3).

### 3.4. Radical-Scavenging Assays

The radical scavenging activity of the essential oil was evaluated using the DPPH and ABTS assays, purchased from Sigma-Aldrich (San Luis, MO, USA), following the procedure described by Cartuche et al. [45], with modifications. Trolox was used as the reference antioxidant, and methanol served as the blank. The results were expressed as SC_50_ values, defined as the essential-oil concentration required to reduce 50% of the initial radical signal. All assays were performed in triplicate.

For both the ABTS and DPPH assays, sample blanks were prepared at each tested essential oil concentration using the corresponding reaction medium without the radical reagent. The absorbance of each sample blank was subtracted from that of the corresponding reaction mixture before calculating the radical-scavenging percentage. This correction was applied to account for potential background absorbance associated with the essential oil, particularly at the higher concentrations evaluated in the DPPH assay.

For both assays, the percentage of radical inhibition was calculated according to the following equation:

$$\mathrm{Inhibition}\left(\%\right)=\frac{A_{0}-A_{s}}{A_{0}}\times 100$$

where *A*_0_ is the absorbance of the radical solution in the absence of the essential oil (control), and *A_s_* is the absorbance of the reaction mixture containing the essential oil. The SC_50_ value was defined as the concentration of essential oil required to reduce 50% of the initial radical signal and was calculated from the corresponding concentration–response curve.

#### 3.4.1. DPPH Radical-Scavenging Assay

For the DPPH assay, a 0.247 mM methanolic solution of 2,2-diphenyl-1-picrylhydrazyl was prepared and adjusted to an absorbance of 1.10 ± 0.01 at 515 nm. Essential-oil solutions were prepared in methanol and tested at final concentrations of 125, 250, 500, 1000, 2000, 4000, and 8000 µg/mL in the DPPH reaction mixture.

For each reaction, 270 µL of the DPPH working solution was mixed with 30 µL of the corresponding essential oil solution in a 96-well microplate. The plate was incubated for 60 min at room temperature in darkness. Absorbance was measured at 515 nm using an Epoch 2 microplate reader (BioTek, Winooski, VT, USA).

The concentration range was selected to encompass the full concentration–response profile of the essential oil, including concentrations producing low radical-scavenging activity, the region around 50% inhibition, and concentrations approaching the maximal response, thereby allowing reliable estimation of the SC_50_.

#### 3.4.2. ABTS Radical-Cation Scavenging Assay

The ABTS radical cation was generated by mixing a 7.4 mM ABTS solution with a 2.6 mM potassium persulfate solution. The mixture was maintained for 16 h at room temperature in darkness. Before analysis, the resulting radical solution was diluted with methanol to obtain an absorbance between 1.15 and 1.20 at 734 nm. Essential oil solutions were prepared in methanol and tested at final concentrations of 3.9, 7.81, 15.62, 31.25, 62.5, 125, and 250 µg/mL in the ABTS reaction mixture. For each reaction, 270 µL of the ABTS working solution was mixed with 30 µL of the corresponding essential-oil solution in a 96-well microplate. The plate was incubated for 60 min at room temperature in darkness, and absorbance was measured at 734 nm using an Epoch 2 microplate reader.

The concentration range was selected to encompass the full concentration–response profile of the essential oil, including concentrations producing low radical-scavenging activity, the region around 50% inhibition, and concentrations approaching the maximal response, thereby allowing reliable estimation of the SC_50_.

### 3.5. Acetylcholinesterase Inhibitory Assay

Acetylcholinesterase (AChE) inhibitory activity was evaluated using an adapted Ellman-based colorimetric microplate assay, originally described by Ellman et al. (1961), with modifications based on previously published procedures for the evaluation of cholinesterase inhibition by essential oils [46,47]. Acetylcholinesterase from Electrophorus electricus (Sigma Aldrich, San Luis, MO, USA) was used as the enzyme source, acetylthiocholine iodide (ATCh) as the substrate, and 5,5′-dithiobis(2-nitrobenzoic acid) (DTNB) as the chromogenic reagent. The final concentrations of ATCh and DTNB in the reaction mixture were approximately 1.41 mM and 1.50 mM, respectively. These conditions represent an adaptation of the original Ellman method to a 96-well microplate format.

The concentration range was selected to characterize the concentration–response relationship of the essential oil and to enable a more reliable estimation of the IC_50_. The essential oil was dissolved in methanol, and working solutions were prepared at concentrations of 10, 200, 500, 1000, and 5000 µg/mL. An aliquot (20 µL) of each working solution was added to the reaction mixture, resulting in final essential oil concentrations of 1, 20, 50, 100, and 500 µg/mL, respectively.

The ATCh solution was prepared by dissolving 12.2 mg of acetylthiocholine iodide in 3 mL of phosphate-buffered saline. The DTNB solution was prepared by dissolving 7.14 mg of DTNB in 6 mL of Tris buffer. The AChE working solution was prepared in Tris buffer at a concentration of 0.5 U/mL.

For each reaction, 100 µL of DTNB solution, 40 µL of phosphate-buffered saline (pH 7.4), 20 µL of the corresponding essential oil working solution, and 20 µL of ATCh solution were added to a 96-well microplate. The mixture was preincubated for 3 min at 37 °C, and the reaction was initiated by adding 20 µL of AChE working solution, resulting in a final reaction volume of 200 µL.

Absorbance at 412 nm was recorded at one-minute intervals over a total monitoring period of 60 min using an Epoch 2 microplate reader (BioTek, Winooski, VT, USA). The absorbance–time profiles were examined to identify the initial approximately linear phase of the enzymatic reaction.

Based on this assessment, the first 10 min of kinetic data were selected for reaction-rate determination. Enzymatic reaction rates were calculated from the slopes obtained by linear regression of absorbance versus time (ΔA/min), rather than from absorbance measurements at a single endpoint.

Sample blanks without AChE were included for each essential oil concentration to account for potential background absorbance and non-enzymatic contributions to the DTNB-based colorimetric reaction. These blanks contained all assay components except AChE, which was replaced with an equal volume of Tris buffer to maintain the final reaction volume of 200 µL.

The absorbance–time slope of each sample blank was subtracted from that of the corresponding reaction mixture to obtain the corrected enzymatic reaction rate.

Methanol was present at a final concentration of 10% (*v*/*v*), and a solvent-matched enzyme control was used as the reference condition. Methanol was used as the negative control, whereas donepezil hydrochloride (Sigma Aldrich, San Luis, MO, USA) was used as the positive reference inhibitor and was assayed under the same experimental conditions.

The percentage of AChE inhibition was calculated from the corrected enzymatic reaction rates relative to the solvent-matched enzyme control. Measurements were performed in triplicate, and the concentration–response data were analyzed by nonlinear regression to estimate the IC_50_ value.

To assess the linearity of the enzymatic reaction during the selected interval, absorbance–time data recorded over the first 10 min were analyzed by linear regression. The analysis included six enzyme-control wells and three replicate wells for each of the five essential oil concentrations tested (1, 20, 50, 100, and 500 µg/mL). The coefficients of determination (R^2^) ranged from 0.9820 to 0.9999. The resulting slopes (ΔA/min) were used to determine the enzymatic reaction rates and calculate the corresponding percentages of AChE inhibition.

### 3.6. Molecular Docking

The 47 compounds identified in the essential oil of *C. chimboracensis* were evaluated against acetylcholinesterase from *E. electricus* (*ee*AChE) to investigate potential molecular contributors to the inhibitory activity observed for the complete oil. The constituents were treated as candidate inhibitory ligands. Molecular docking was used to predict their positions, orientations, and conformations within the enzyme’s binding cavity. The AlphaFold Protein Structure Database (EMBL-EBI, Hinxton, UK) [48] model of *ee*AChE corresponding to UniProt accession O42275 was used as the receptor after removal of the low-confidence N-terminal segment comprising residues 1–28 and the C-terminal segment comprising residues 593–633.

For each compound, an initial three-dimensional conformation was generated from its simplified molecular input line entry system (SMILES) representation using OpenEye OMEGA 6.1.1.1 (OpenEye, Cadence Molecular Sciences, Santa Fe, NM, USA) [49,50], a molecular conformation-generation program. Classic mode was used by default, whereas Macrocycle mode was used for molecules containing rings with 10 or more members. Partial atomic charges were subsequently assigned using the Austin Model 1 with bond-charge corrections (AM1-BCC) method implemented in QUACPAC 2.1.1.2 OpenEye, Cadence Molecular Sciences, Santa Fe, NM, USA) [51]. This method combines charges derived from a semiempirical quantum-mechanical calculation with corrections associated with chemical bonds to represent the molecular charge distribution.

The ligand binding cavity in *ee*AChE was located using the donepezil-bound human acetylcholinesterase structure (*hs*AChE; PDB 4EY7) as a reference. The processed O42275 model was structurally aligned to 4EY7, and the ligand coordinate positions of the reference structure were transferred to the *ee*AChE model to locate the corresponding binding region. Among the 32 corresponding residues selected around this region, 30 were identical between eeAChE and hsAChE (93.75% identity). The selected eeAChE residues had a mean pLDDT of 96.72 (range, 87.25–98.94), with 30 residues scoring above 90, indicating high local prediction confidence. A local least-squares fit of the 32 C*α* pairs yielded an RMSD of 0.59 Å, supporting a conserved backbone arrangement among the selected residues (Figure S3 in Supplementary Materials).

Molecular docking calculations were performed with GOLD 2025.5 (Genetic Optimisation for Ligand Docking; Cambridge Crystallographic Data Centre, Cambridge, UK) [52]. GOLD uses a genetic algorithm to explore alternative ligand arrangements within a defined protein binding region. The docking cavity was then defined as a 6 Å radius region centered on the transferred ligand position. Calculations were conducted with the ChemPLP, a scoring function combining a piecewise linear potential for steric contacts with hydrogen-bonding terms. Ligands were treated as fully flexible, and 30 poses were generated for each acetylcholinesterase–ligand pair. The search efficiency was set to 200%, and early termination was disabled.

The resulting poses were rescored with the GoldScore, ChemScore, and Astex Statistical Potential (ASP) scoring functions from GOLD (Cambridge Crystallographic Data Centre, Cambridge, UK). These functions provide complementary assessments of the predicted protein–ligand arrangements. Scores from these functions and the primary ChemPLP function were standardized as Z-scores within each compound to allow their combination across different numerical scales. An aggregated consensus score (AggScore) was calculated as the average Z-score across the scoring functions, as previously described [53]. All poses with AggScore > 1 were selected for subsequent absolute binding free-energy (ABFE) calculations. For compounds without a pose exceeding this threshold, the pose with the highest AggScore was retained. These docking scores were used for pose selection and were not interpreted as absolute binding free energies.

### 3.7. Absolute Binding Free Energy Calculations from Molecular Dynamics Simulations

BAT.py v2.4 (Germano Heinzelmann, Universidade Federal de Santa Catarina, Florianópolis, Brazil) [12] was used to perform absolute binding free energy (ABFE) calculations, with Amber26 (University of California, San Francisco, San Francisco, CA, USA) [54] as the simulation engine. For the acetylcholinesterase receptor, the hsAChE structure (PDB ID 4EY7) was first used to generate the reference structure file and receptor-specific parameters required by BAT.py.

Because the simultaneous decoupling and recoupling (SDR) protocol separates the bound ligand and the ligand copy in bulk solvent along the z axis, the reference receptor was oriented with its longest dimension approximately parallel to this axis. In this work, the orientation was defined using the C*α* atoms of the protein.

The sdr_dist parameter was defined by translating the bound ligand along the positive z direction until the minimum distance between the displaced ligand and any protein atom was at least 15 Å. The final sdr_dist value was calculated as the distance between the centers of mass of the bound ligand and the translated ligand copy.

Binding cavities were identified with the CASTpFold web server [55], and the center of the binding pocket was determined from the geometric centroid of its assigned atoms. A tentative ligand L1 anchor was assigned as the reference ligand atom closest to this center. Receptor anchors P1, P2, and P3 were selected from backbone atoms located in stable secondary-structure elements, avoiding loop regions. P1 was chosen at a distance of 7–12 Å from L1 and with the P1–L1 vector oriented as parallel as possible to the z axis, whereas P2 and P3 were selected so that the L1–P1–P2 and P1–P2–P3 angles were close to 90° and the P1–P2 and P2–P3 distances were at least 8 Å. The BAT variables l1_x, l1_y, and l1_z were defined from the Cartesian components of the P1–L1 vector, and l1_range was defined as the maximum distance between L1 and any atom assigned to the selected cavity.

BAT.py calculations were run in “crystal” mode starting from the docking-selected complexes. ABFE values were estimated from restrained and alchemically transformed molecular dynamics simulations using the express protocol of BAT.py. Systems were parametrized with the ff19SB forcefield for the receptor and GAFF2 for the ligands. Simulations were carried out with the OPC water model, Na^+^ and Cl^−^ ions at 0.15 M, hydrogen mass repartitioning, a temperature of 298.15 K, a 9.0 Å nonbonded cutoff, Langevin dynamics with gamma_ln = 1.0 ps^−1^, a Monte Carlo barostat, and a 4 fs time step.

Equilibration was performed with gradual release of ligand restraints using weights of 10.0, 2.5, 0.5, and 0.0, with 100,000 steps per release stage, followed by 1,000,000 additional equilibration steps. Free energy calculations were carried out by Thermodynamic Integration (TI) with Gaussian quadrature using 12 TI points and five blocks for block analysis. The translational and rotational restraint component and the ligand conformational restraint component were run with 25,000 equilibration and 50,000 production steps per window. The electrostatic decoupling and recoupling component included 50,000 equilibration and 50,000 production steps per window, whereas the van der Waals decoupling and recoupling component consisted of 100,000 equilibration and 100,000 production steps per window. The restraint force constants were 5.0 kcal·mol^−1^·Å^−2^ for the ligand distance restraint, 250.0 kcal·mol^−1^·rad^−2^ for ligand angular and dihedral restraints, 30.0 kcal·mol^−1^·rad^−2^ for ligand conformational dihedral restraints, and 10.0 and 5.0 kcal·mol^−1^·Å^−2^ for the receptor and bulk-ligand center-of-mass restraints, respectively. Binding free energies were reported as ΔG ± SEM in kcal/mol.

Within-run sampling stability and numerical uncertainty were assessed with the block-analysis procedure implemented in BAT.py. Production data from every simulation window were divided chronologically into five equal-duration blocks. Each free-energy component *j* was recalculated from each block using the same estimator used for the complete production trajectory. BAT.py calculates the population standard deviation across the five block-specific estimates *σ_j_*_,*block*_ as:

$$\sigma_{j,\mathrm{block}}=\sqrt{\frac{1}{5}\sum_{b=1}^{5}{\left(\Delta G_{j,b}-{\bar{\Delta G}}_{j,\mathrm{blocks}}\right)}^{2}},$$

where

$${\bar{\Delta G}}_{j,\mathrm{blocks}}=\frac{1}{5}\sum_{b=1}^{5}\Delta G_{j,b}$$

The standard error of the mean (SEM) for the total binding free energy defined for BAT was obtained by adding the block dispersions of the simulated free-energy components in quadrature:

$$\mathrm{SEM}=\sqrt{\sum_{j}\sigma_{j,\mathrm{block}}^{2}}$$

### 3.8. Statistical Analysis

All antioxidant activity and acetylcholinesterase inhibition measurements were performed in three technical replicates for each tested concentration using the same essential-oil preparation; therefore, the triplicate measurements represent technical replicates rather than independent experiments. Data are expressed as mean ± standard deviation (SD), and the standard error of the mean (SEM) was calculated as SD/√n, where n = 3 technical replicates. Concentration–response data were analyzed using GraphPad Prism version 11.1.0 (GraphPad Software, Boston, MA, USA). Concentration–response curves were fitted by nonlinear regression using a four-parameter logistic model with variable slope. SC_50_ values for the ABTS and DPPH assays and IC_50_ values for the AChE inhibition assay were estimated from the corresponding fitted concentration–response curves. The goodness of fit of the nonlinear regression models was assessed using the coefficient of determination (R^2^), and Hill slope values were also reported. SC_50_ and IC_50_ estimates are presented with their corresponding 95% confidence intervals.

## 4. Conclusions

1. The leaf essential oil of *C. chimboracensis* contained 47 identified volatile constituents, accounting for 95.17% of the total GC–FID peak area. Sesquiterpene hydrocarbons predominated, with *trans*-muurola-4(14),5-diene, longifolene, bicyclogermacrene, caryophyllene oxide, *α*-humulene, and *γ*-cadinene as the principal constituents.
2. The essential oil exhibited an assay-dependent radical-scavenging response, with SC_50_ values of 15.20 µg/mL in the ABTS assay and 2983 µg/mL in the DPPH assay. These findings describe its radical-scavenging activity under the experimental conditions employed.
3. The complete essential oil inhibited acetylcholinesterase from *E. electricus*, with an IC_50_ value of 43.78 µg/mL (95% confidence interval: 40.68–47.05 µg/mL), with a Hill slope of −1.801 and a coefficient of determination (R^2^) of 0.9927. This result establishes the inhibitory activity of the mixture without assigning the measured response to individual constituents.
4. Molecular docking followed by absolute binding free-energy calculations prioritized spirolepechinene, *β*-phellandrene, *β*-pinene, alloaromadendrene epoxide, and (2*E*,4*E*)-nona-2,4-dien-1-ol as potential AChE-binding constituents. These predictions provide molecular hypotheses for subsequent experimental validation but do not establish individual inhibitory potency or synergistic interactions.
5. The integrated chemical, biological, and computational characterization provides a basis for further investigation of *C. chimboracensis* essential oil. Future studies should evaluate the prioritized constituents individually and in defined mixtures to determine their contributions to the observed AChE inhibition. Neuroprotective activity was not evaluated in this study and requires separate investigation in appropriate cellular and biological models.

## Figures and Tables

**Table 1:** Volatile constituents identified in the leaf essential oil of *C. chimboracensis* by GC–MS and their relative composition determined by GC–FID peak-area normalization on a TR-5MS column.

N°	Compound	LRI ^a^	LRI ^b^	Relative Area (%) ^c^	Molecular Formula
1	Tricyclene	922	921	0.07 ± 0.001	C_10_H_16_
2	*α*-Thujene	925	924	0.19 ± 0.006	C_10_H_16_
3	*α*-Pinene	933	932	2.16 ± 0.046	C_10_H_16_
4	Camphene	950	946	0.48 ± 0.009	C_10_H_16_
5	Sabinene	974	969	0.22 ± 0.011	C_10_H_16_
6	*β*-Pinene	979	974	1.57 ± 0.029	C_10_H_16_
7	Myrcene	991	988	1.47 ± 0.027	C_10_H_16_
8	*ρ*-Cymene	1029	1020	0.02 ± 0.001	C_10_H_14_
9	Limonene	1032	1024	1.26 ± 0.031	C_10_H_16_
10	*β*-Phellandrene	1034	1025	1.80 ± 0.039	C_10_H_16_
11	*γ*-Terpinene	1061	1054	0.11 ± 0.003	C_10_H_16_
12	*cis*-Chrysanthenol	1146	1160	0.04 ± 0.001	C_10_H_16_O
13	Borneol	1183	1165	0.10 ± 0.002	C_10_H_18_O
14	(2*E*,4*E*)-nona-2,4-dien-1-ol	1201	1217	0.13 ± 0.021	C_9_H_16_O
15	Safranal	1211	1197	0.25 ± 0.010	C_10_H_14_O
16	*trans*-Chrysanthenyl acetate	1236	1235	1.22 ± 0.034	C_12_H_18_O_2_
17	Bornyl acetate	1291	1284	1.17 ± 0.016	C_12_H_20_O_2_
18	*trans*-Pinocarvyl acetate	1303	1298	0.17 ± 0.005	C_12_H_18_O_2_
19	Presilphiperfol-7-ene	1347	1334	0.05 ± 0.001	C_15_H_24_
20	*α*-Ylangene	1377	1373	1.59 ± 0.021	C_15_H_24_
21	*β*-Bourbonene	1385	1387	0.94 ± 0.119	C_15_H_24_
22	Linalool isobutanoate	1391	1373	0.48 ± 0.088	C_14_H_24_O_2_
23	iso-Italicene	1400	1401	0.08 ± 0.003	C_15_H_24_
24	Longifolene	1422	1407	15.34 ± 0.398	C_15_H_24_
25	Aromadendrene	1433	1439	0.25 ± 0.016	C_15_H_24_
26	Guaiadiene <6,9->	1444	1442	0.62 ± 0.018	C_15_H_24_
27	Spirolepechinene	1448	1449	0.35 ± 0.333	C_15_H_24_
28	*α*-Humulene	1460	1452	4.38 ± 0.208	C_15_H_24_
29	y-Muurolene	1480	1478	0.51 ± 0.016	C_15_H_24_
30	*trans*-Muurola-4(14),5-diene	1487	1493	34.06 ± 0.832	C_15_H_24_
31	10,11-epoxy-Calamenene	1497	1491	0.77 ± 0.022	C_15_H_20_O
32	Bicyclogermacrene	1501	1500	7.65 ± 0.330	C_15_H_24_
33	(*E*,*E*)- *α*-Farnesene	1511	1505	0.10 ± 0.002	C_15_H_24_
34	*γ*-Cadinene	1523	1513	4.02 ± 0.086	C_15_H_24_
35	*α*-Calacorene	1550	1544	0.85 ± 0.021	C_15_H_20_
36	Spathulenol	1560	1577	0.33 ± 0.009	C_15_H_24_O
37	Germacrene B	1566	1559	1.73 ± 0.043	C_15_H_24_
38	*β*-Calacorene	1572	1564	0.12 ± 0.002	C_15_H_20_
39	*β*-Atlantol	1589	1608	0.12 ± 0.001	C_15_H_24_O
40	Caryophyllene oxide	1593	1582	5.53 ± 0.445	C_15_H_24_O
41	*α*-Acorenol	1638	1632	0.23 ± 0.072	C_15_H_26_O
42	Alloaromadendrene epoxide	1646	1639	0.15 ± 0.066	C_15_H_24_O
43	*α*-Muurolol	1654	1644	0.97 ± 0.265	C_15_H_26_O
44	*α*-Cadinol	1669	1652	0.96 ± 0.060	C_15_H_26_O
45	Valerianol	1673	1656	0.29 ± 0.044	C_15_H_26_O
46	*trans*-Calamenen-10-ol	1681	1668	0.16 ± 0.167	C_15_H_22_O
47	Cadalene	1687	1675	0.11 ± 0.075	C_15_H_18_
	**Hydrocarbon monoterpenes**			9.35	
	**Oxygenated monoterpenes**			0.39	
	**Hydrocarbon sesquiterpenes**			72.75	
	**Oxygenated sesquiterpenes**			9.51	
	**Other compounds**			3.17	
	**Total**			95.17	

LRI ^a^, calculated linear retention index; LRI ^b^, the literature linear retention index [13]; ^c^ values are expressed as mean relative peak area (%) ± standard deviation from three independent distillations (*n* = 3).

**Table 2:** Radical-scavenging activity and Trolox equivalent antioxidant capacity of the leaf essential oil of *Croton chimboracensis*.

Assay	SC_50_ (µg/mL)	95% CI (µg/mL)	Hill Slope	R^2^	TEAC µmol Trolox Equivalents/g EO
ABTS	15.20	8.97–19.83	1.345	0.9820	21.53 ± 1.79
DPPH	2983	2719–3378	1.855	0.9971	-

Trolox was used as the reference antioxidant. SC_50_ values are reported with their 95% confidence intervals, obtained by profile-likelihood analysis. R^2^ represents the coefficient of determination of the four-parameter logistic nonlinear regression model. TEAC values are expressed as the mean ± SD (n = 3).

## Data Availability

All data generated or analyzed during this study are included within this article.

## Supplementary Materials

The following supporting information can be downloaded at: https://www.mdpi.com/article/10.3390/plants15213481/s1, Figure S1: Chemical structures of the 47 compounds identified in the essential oil of *C. chimboracensis*; Table S1: Individual replicate values and descriptive statistics for the ABTS and DPPH radical-scavenging assays of the leaf essential oil of *C. chimboracensis*; Figure S2: Absorbance–time curves at 412 nm during the acetylcholinesterase inhibition assay; Table S2: Linear regression parameters of the absorbance–time curves at 412 nm for the enzymatic control and the concentrations evaluated in the acetylcholinesterase inhibition assay; Table S3: Nonlinear regression parameters of the concentration–response curve for acetylcholinesterase inhibition using a sigmoidal dose–response model; Table S4: SMILES strings for the compounds identified in the essential oil of *C. chimboracensis;* Table S5: Docking results for each compound evaluated against eeAChE; Table S6: ABFE results for all evaluated protein–ligand complexes; Figure S3: Structural comparison of the donepezil-binding region in eeAChE and hsAChE.
