# Supplementary material for: Chemical Composition, Antioxidant, and Antiacetylcholinesterase Activities of the Essential Oil of Croton chimboracensis: In Vitro and In Silico Approaches
Source: Plants (Basel). 2026 Nov 7;15(21):3481. doi: 10.3390/plants15213481 (PMC13765981; doi:10.3390/plants15213481)
Supplement: Supplementary file 1 [file plants-15-03481-s001.zip › plants-4641046-supplementary.pdf]

Supplementary Materials

Figure S1. Chemical structures of the 47 compounds identified in the essential oil of *Croton chimboracensis*. Compounds numbering corresponds to Table 1.

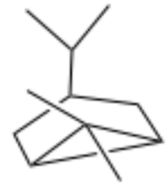

1

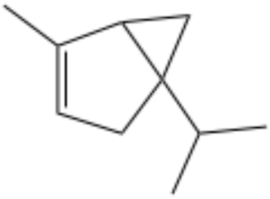

2

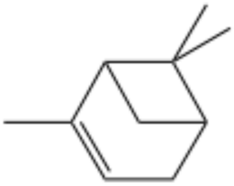

3

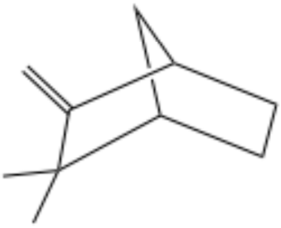

4

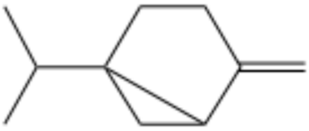

5

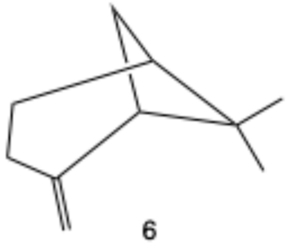

6

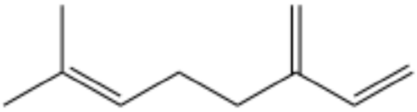

7

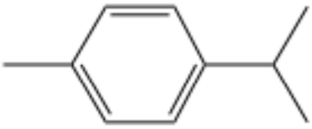

8

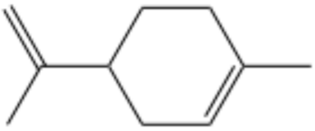

9

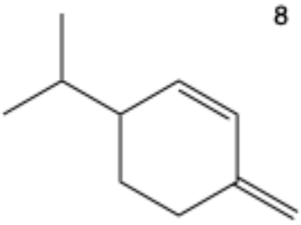

10

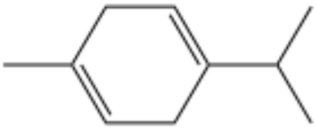

11

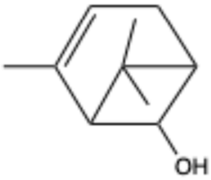

12

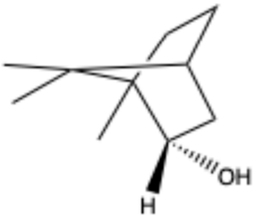

13

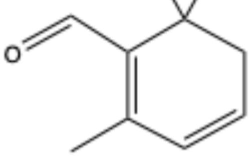

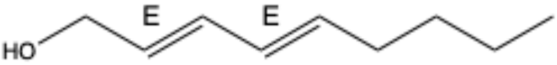

14

15

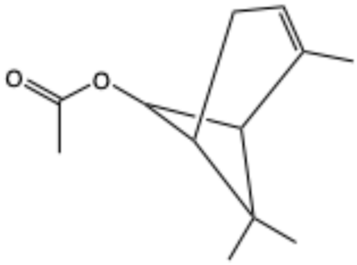

16

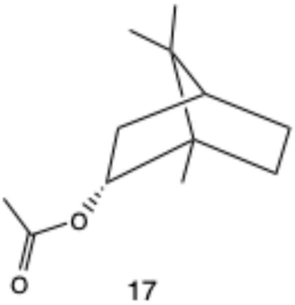

17

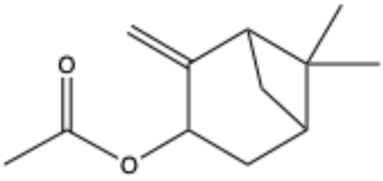

18

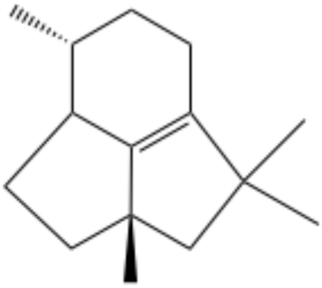

19

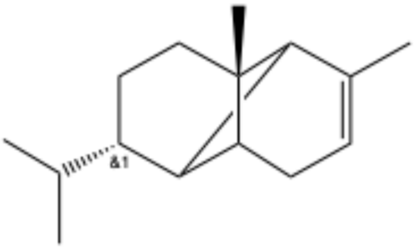

20

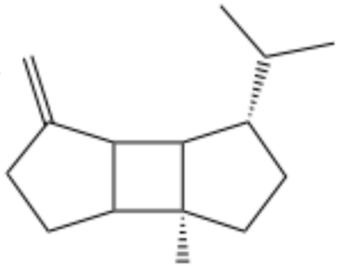

21

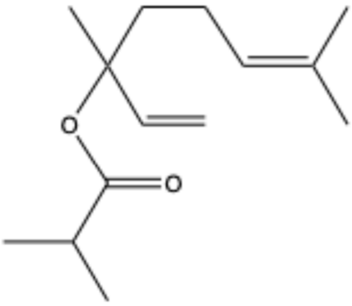

22

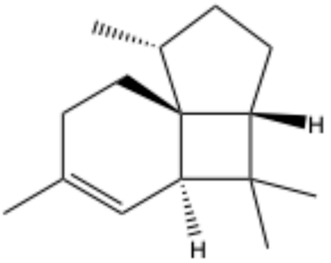

23

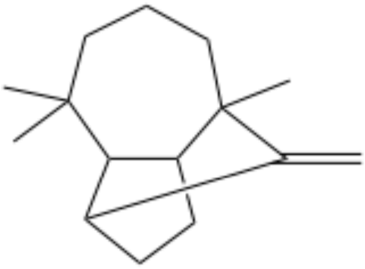

24

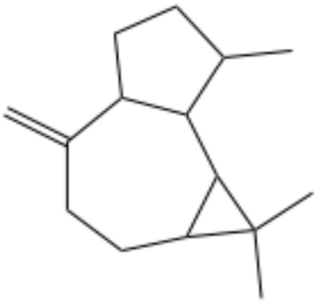

25

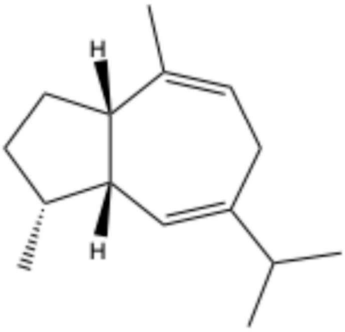

26

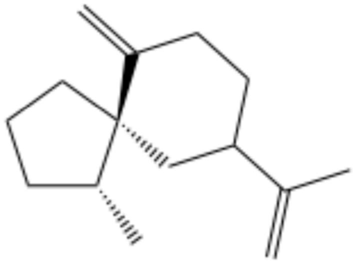

27

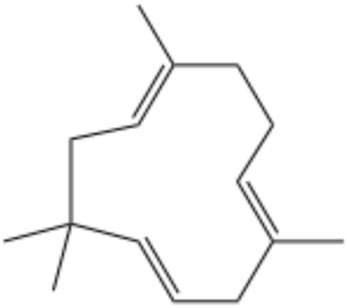

28

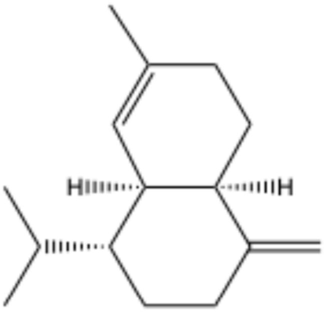

29

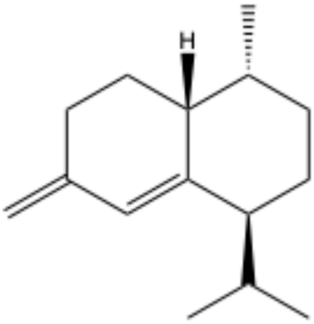

30

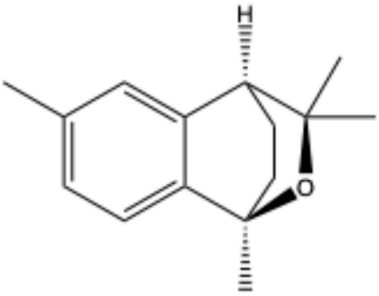

31

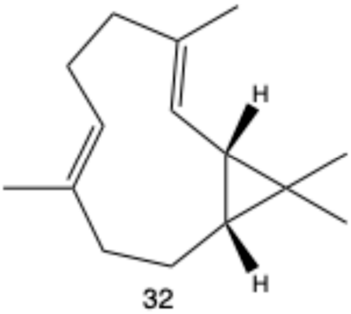

32

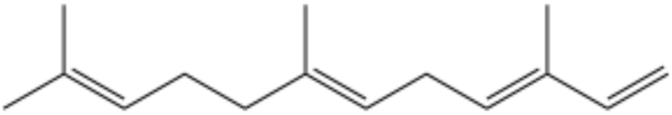

33

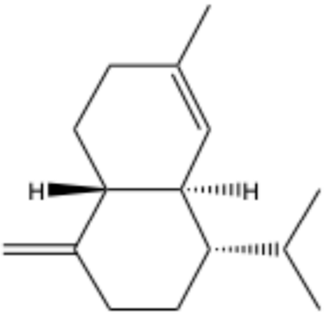

34

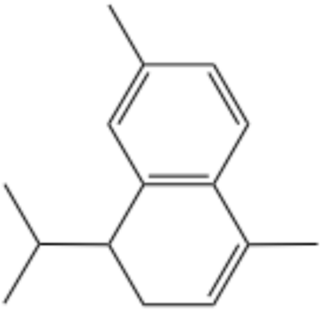

35

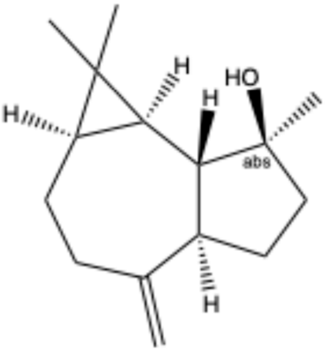

36

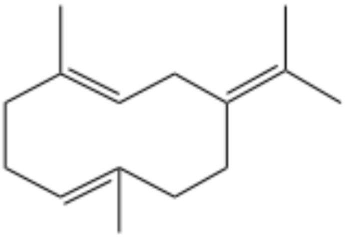

37

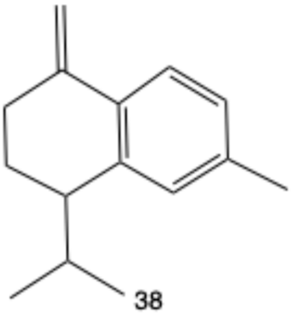

38

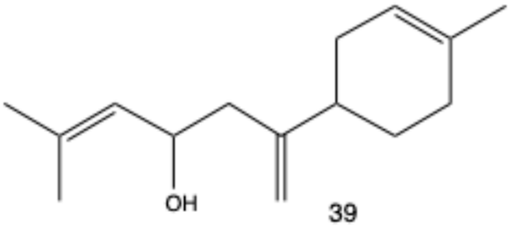

39

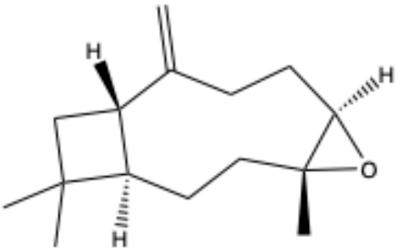

40

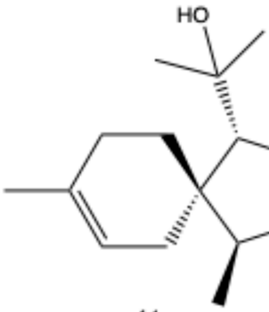

41

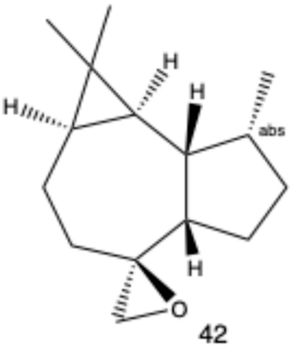

42

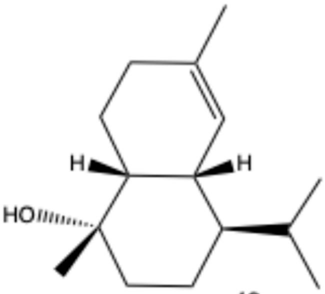

43

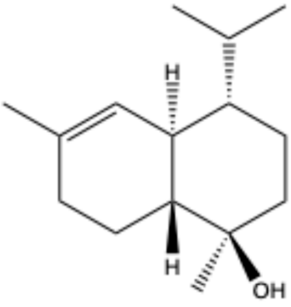

44

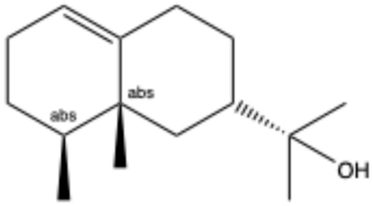

45

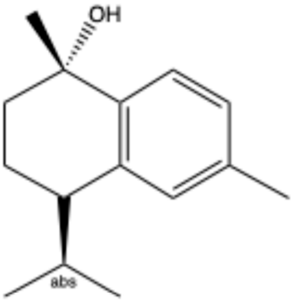

46

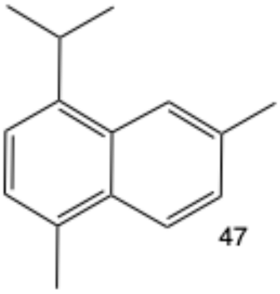

47

Table S1. Individual replicate values and descriptive statistics for the ABTS and DPPH radical-scavenging assays of the leaf essential oil of *C. chimboracensis*.

Assay	Final concentration (µg/mL	Replicate 1 (%)	Replicate 2 (%)	Replicate 3 (%)	Mean (%)	SD (%)	SEM (%)
ABTS	3.9	11.9	11.73	11.873	11.834	0.091	0.053
	7.81	23.86	24.29	24.003	24.051	0.219	0.126
	15.62	53.65	54.35	53.463	53.821	0.468	0.27
	31.25	62.32	64.49	62.437	63.082	1.220	0.705
	62.5	87.96	89.86	88.937	88.919	0.95	0.549
	125	92.63	92.37	92.573	92.524	0.137	0.079
	250	92.72	92.89	92.690	92.767	0.108	0.062
DPPH	125	3.026	2.737	3.479	3.080	0.374	0.216
	250	5.299	5.897	5.812	5.670	0.324	0.187
	500	8.615	8.205	8.034	8.285	0.299	0.172
	1000	15.983	14.872	20.171	17.009	2.794	1.613
	2000	32.393	32.991	34.017	33.134	0.821	0.474
	4000	64.957	67.094	66.410	66.154	1.091	0.63
	8000	85.128	86.581	86.923	86.211	0.953	0.55

Values represent radical-scavenging activity (%) at each final essential-oil concentration. Measurements were performed in triplicate (n = 3). SD, standard deviation; SEM, standard error of the mean. SEM was calculated as SD/√n

Figure S2. **Absorbance–time curves at 412 nm during the acetylcholinesterase inhibition assay.**

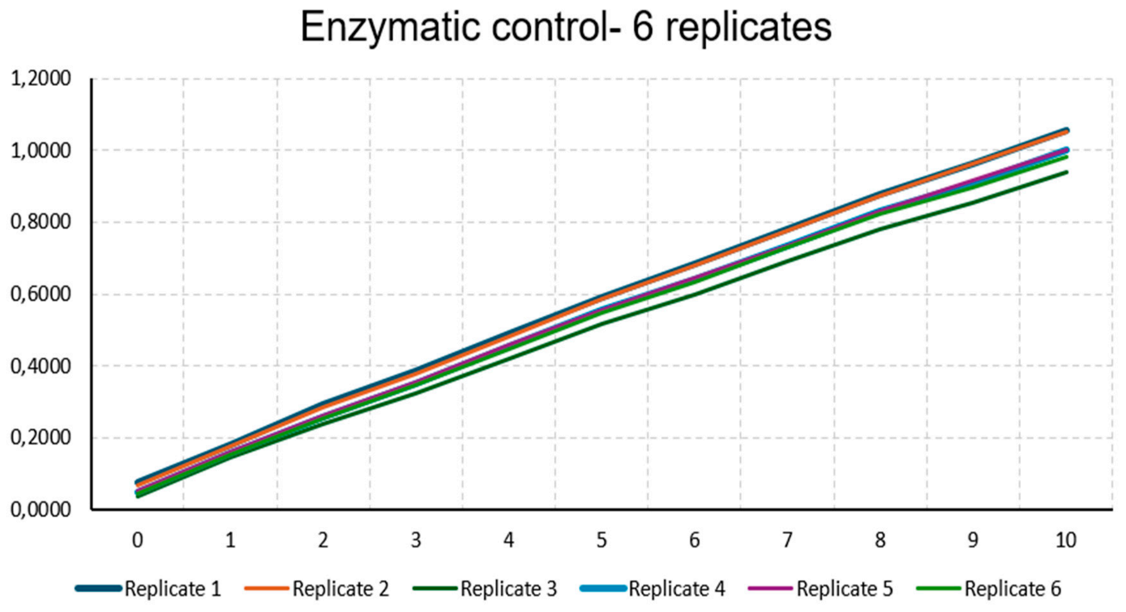

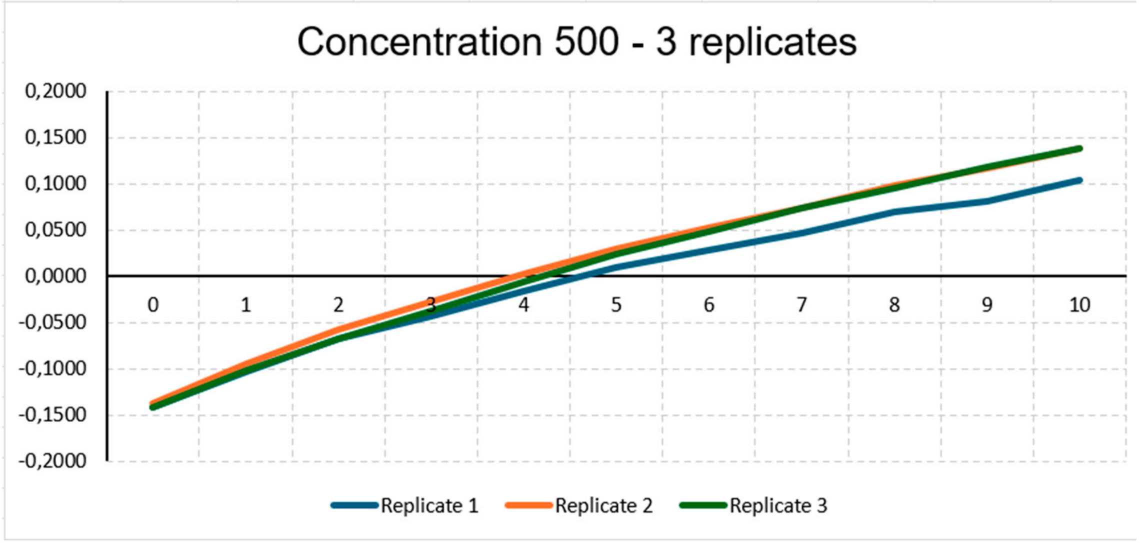

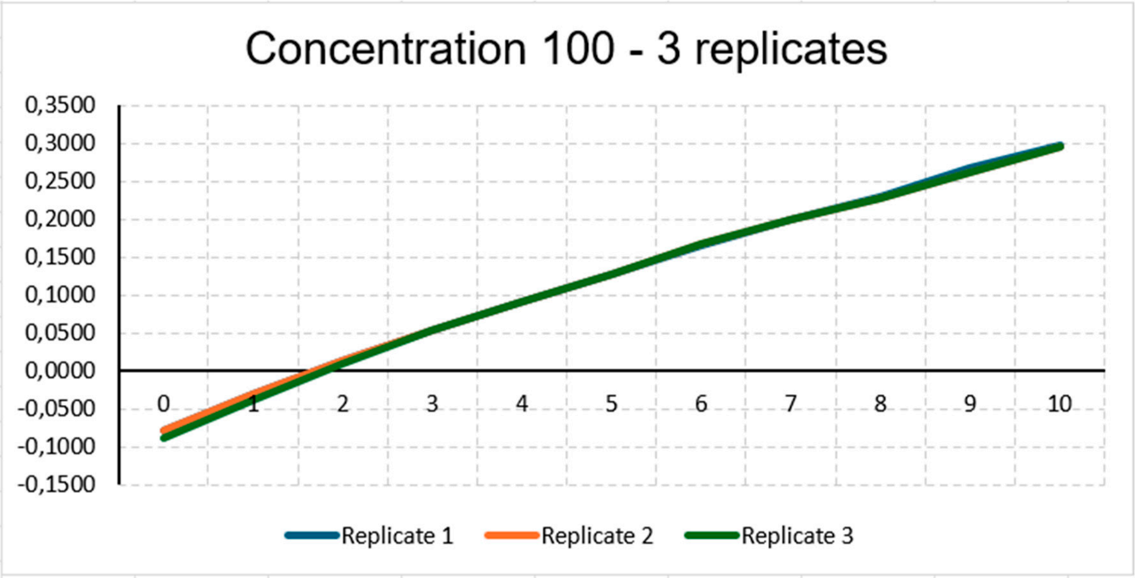

Concentration 100 - 3 replicates
0,3500
0,3000
0,2500
0,2000
0,1500
0,1000
0,0500
0,0000
-0,0500
-0,1000
-0,1500
0
1
2
3
4
5
6
7
8
9
10
Replicate 1
Replicate 2
Replicate 3

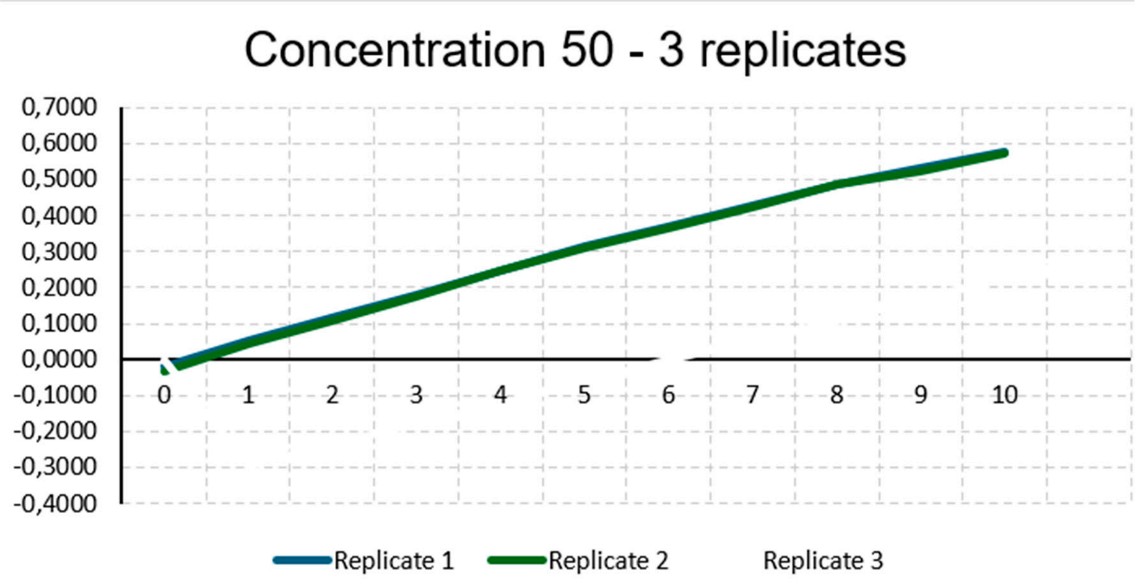

Concentration 50 - 3 replicates
0,7000
0,6000
0,5000
0,4000
0,3000
0,2000
0,1000
0,0000
-0,1000
-0,2000
-0,3000
-0,4000
0
1
2
3
4
5
6
7
8
9
10
Replicate 1
Replicate 2
Replicate 3

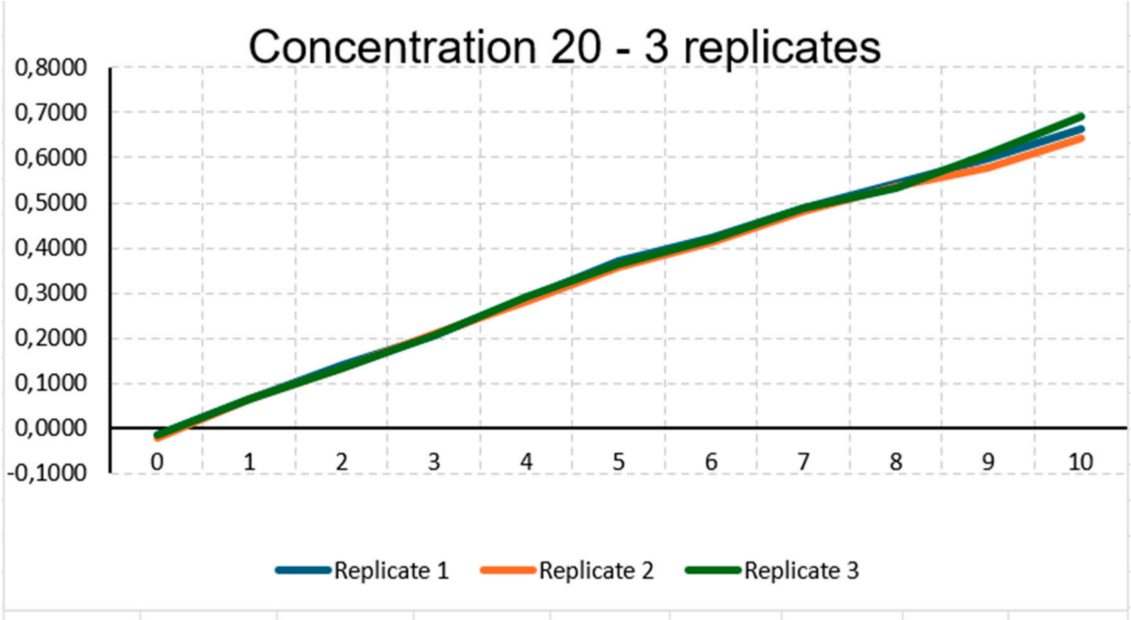

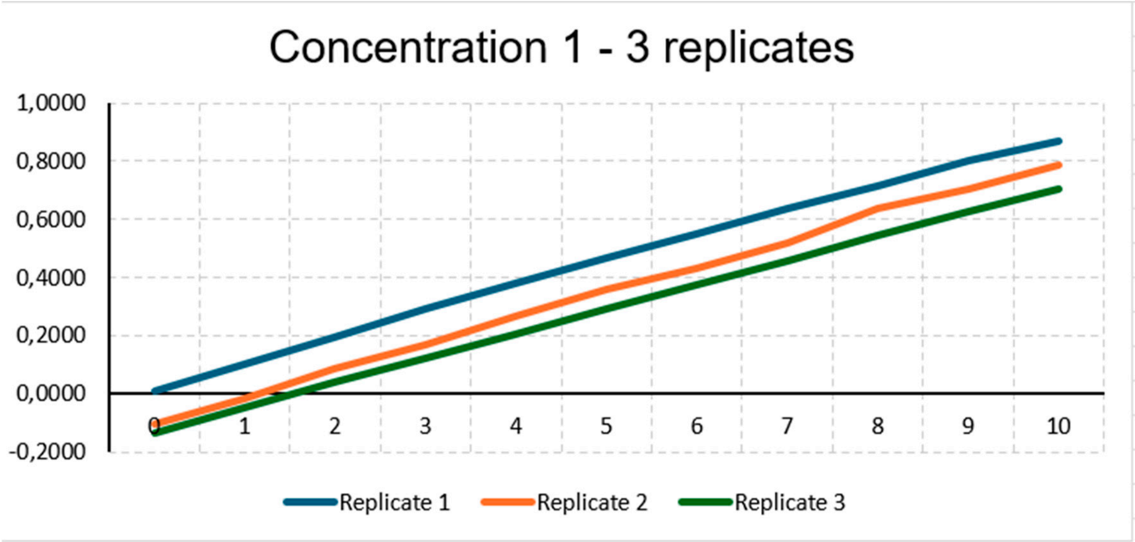

Note. Absorbance–time curves recorded at 412 nm during the first 10 minutes of the acetylcholinesterase (AChE) inhibition assay using the modified Ellman method. The panels represent (A) the enzymatic control (n = 6) and the tested concentrations of (B) 1, (C) 20, (D) 50, (E) 100, and (F) 500 µg/mL (n = 3 per concentration). Absorbance was measured at 1-minute intervals to assess reaction linearity and determine enzymatic reaction rates from the slopes of the linear portions of the curves.

Table S2. Linear regression parameters of absorbance–time curves at 412 nm for the enzymatic control and different concentrations evaluated in the acetylcholinesterase (AChE) inhibition assay.

Condicion	Pocillo	Pendiente_Abs	Intercepto	R2	N_lecturas
Control	A4	0,097981818	0,089631184	0,99920817	11
Control	A5	0,098363636	0,082813003	0,999219384	11
Control	A6	0,089718182	0,055131184	0,998970856	11
Control	A7	0,094836364	0,065449366	0,998930671	11
Control	A8	0,094790909	0,068858457	0,999036904	11
Control	A9	0,0939	0,063131184	0,998469291	11
500 ug/mL	D4	0,023609091	-0,12105063	0,982033402	11
500 ug/mL	D5	0,026690909	-0,11582336	0,985097537	11
500 ug/mL	D6	0,027690909	-0,12564154	0,990126181	11
100 ug/mL	D7	0,037190909	-0,06495973	0,996446824	11
100 ug/mL	D8	0,036863636	-0,06414154	0,995665956	11
100 ug/mL	D9	0,037736364	-0,07059609	0,993460703	11
50 ug/mL	D10	0,060318182	-0,00359609	0,995826183	11
50 ug/mL	D11	0,057336364	-0,29223245	0,999880577	11
50 ug/mL	D12	0,060945455	-0,01173245	0,995070128	11
20 ug/mL	C1	0,067545455	0,00508573	0,995574415	11
20 ug/mL	C2	0,066027273	0,004494821	0,994521093	11
20 ug/mL	C3	0,068881818	-5,0634E-05	0,997049175	11
1 ug/mL	C4	0,086381818	0,024813003	0,998752931	11
1 ug/mL	C5	0,089345455	-0,09791427	0,999077812	11
1 ug/mL	C6	0,0837	-0,12814154	0,999942541	11

Note: Linear regression analysis was performed using absorbance measurements recorded at 412 nm over a 10-minute interval (0–10 min), with 11 measurements per replicate. The table presents the slope (ΔA/min), y-intercept, and coefficient of determination (R^2) for each individual replicate. Six replicates were analyzed for the enzymatic control (n = 6), and three replicates were analyzed for each tested concentration (1, 20, 50, 100, and 500 µg/mL; n = 3). The slopes represent the rates of change in absorbance over time and were used to estimate enzymatic reaction rates.

Table S3. Nonlinear regression parameters of the concentration–response curve for acetylcholinesterase (AChE) inhibition using a sigmoidal dose–response model.

Nonlinear regression parameters and goodness-of-fit statistics for AChE inhibition		
**Parameter**	**Estimated value**	**95% Confidence Interval (CI)**
IC50 (µg/mL)	43,78	40.68–47.05
log10(IC50)	1,641	1.609–1.673
Hill slope	-1,801	−2.055 to −1.580
Coefficient of determination (R^2)	0,9927	—
Residual standard deviation (Sy.x)	3,423	—
Residual sum of squares	152,4	—
Degrees of freedom	13,	—
Lack-of-fit test (F)	0,1061	—
Lack-of-fit p-value	0,9546	—
Number of observations (n)	15,	—
Note: 95% CIs were calculated by profile likelihood; variable-slope nonlinear regression (log[inhibitor] vs. normalized response).		

Note: Nonlinear regression analysis was performed using GraphPad Prism 11 to fit the concentration–response data to a four-parameter logistic (4PL) model. The table presents the half-maximal inhibitory concentration (IC_{50}), its 95% confidence interval (95% CI), the Hill slope, and the coefficient of determination (R^2). Model adequacy was assessed using the lack-of-fit test, when applicable. The IC_{50} represents the concentration producing 50% of the fitted maximal inhibitory effect, while the Hill slope describes the steepness of the concentration–response relationship.

Table S4. SMILES code for each *C. chimboracensis* essential oil compounds

**Compound name**	**SMILES**
Thujene < α->	CC(C12CC=C(C)C1C2)C
Tricyclene	C12C(CC(C(C)C)C1)C2(C)C
Pinene <α->	C1(C)C2CC(C2(C)C)CC=1
Camphene	C1(C(C2CCC1C2)=C)(C)C
Sabinene	C12(C(C(=C)CC1)C2)C(C)C
Pinene <β->	CC1(C2CCC(=C)C1C2)C
Myrcene	C=CC(=C)CC/C=C(\C)/C
Cymene <ϱ->	C1C=C(C(C)C)C=CC=1C
Limonene	C(C1CC=C(CC1)C)(=C)C
Phellandrene <β->	C1C(C(C)C)CCC(=C)C=1
Terpinene <y>	C1C(C)=CCC(C(C)C)=C1
Chrysanthenol <cis->	C1(O)C2C(C)(C)C1C(C)=CC2
Borneol	C1C2C(C)(C)C(C)(C1)[C@](O)([H])C2
(2E,4E)-nona-2,4-dien-1-ol	C(O)/C=C/C=C/CCCC
Safranal	C(C1=C(C=CCC1(C)C)C)=O
Chrysanthenyl acetate <trans->	C(=O)(C)OC1C2CC=C(C)C1C2(C)C
Bornyl acetate	C12(CCC(C[C@H]1OC(=O)C)C2(C)C)C
Pinocarvyl acetate <trans->	C(OC1CC2CC(C2(C)C)C1=C)(=O)C
Presilphiperfol-7-ene	[C@@H]1(C)CCC2=C3[C@@](C)(CCC13)CC2(C)C
Ylangene <α->	[C@H]1(C(C)C)C2C3C(C)=CCC2[C@]3(C)CC1
Bourbonene <β->	C12[C@]3(C)CC[C@@H](C(C)C)C3C1C(=C)CC2
Linalool isobutanoate	C(/C=C(\C)/C)CC(OC(=O)C(C)C)(C)C=C
Italicene <iso->	[C@@]123[C@H](C)CC[C@]1([H])C(C)(C)[C@@]2([H])C=C(C)CC3
Longifolene	C1(CCCC2(C)C(=C)C3CCC2C13)(C)C
Aromadendrene	C1(C2CCC(=C)C3CCC(C)C3C12)(C)C
Guaiadiene <6,9->	[C@H]1(C)[C@]2([H])[C@@]([H])(C(C)=CCC(C(C)C)=C2)CC1
Spirolepechinene	[C@H]1(C)[C@@]2(CC(C(C)=C)CCC2=C)CCC1
Humulene <α->	CC1=CCCC(=CCC(C)(C)C=CC1)C
Muurolene <y>	C([C@H]1CCC(=C)[C@]2([H])CCC(C)=C[C@]12[H])(C)C
Muurola-4(14),5-diene <trans->	[H][C@@]12[C@@H](CC[C@H](C1=CC(CC2)=C)C(C)C)C
Calamenene <10,11-epoxy->	[H][C@@]12[C@](O[C@@](C3C1=CC(=CC=3)C)(CC2)C)(C)C
Bicyclogermacrene	CC1=C[C@H]2[C@@H](CCC(=CCC1)C)C2(C)C
Farnesene <(E,E)- α->	C=C/C(/C)=C/C/C=C(\C)/CC/C=C(\C)/C
Cadinene <γ->	C1(C)=C[C@@]2([H])[C@H](C(C)C)CCC(=C)[C@]2([H])CC1
Calacorene <α->	C1(C)C=CC2C(C)=CCC(C(C)C)C=2C=1
Spathulenol	C1[C@]2([H])[C@]([H])([C@]3([H])C(C)(C)[C@]3([H])CCC2=C)[C@@](C)(O)C1

Germacrene B	CC(C)=C1CC=C(C)CCC=C(C)CC1
Calacorene <β->	C1(=C)C2C=CC(C)=CC=2C(C(C)C)CC1
Atlantol <β->	C1(C(=C)CC(/C=C(/C)\C)O)CCC(C)=CC1
Caryophyllene oxide	[C@@H]23CC[C@]1(O[C@@H]1CCC(=C)[C@H]2CC3(C)C)C
Acorenol <α->	[C@H]1(C(C)(C)O)[C@]2(CCC(C)=CC2)[C@H](C)CC1
Aromadendrene epoxide <allo->	C1[C@@]2([H])[C@]([H])([C@]3([H])C(C)(C)[C@]3([H])CC[C@]32CO3)[C@H](C)C1
Muurolol <α->	C1C[C@](C)(O)[C@]2([H])CCC(C)=C[C@]2([H])[C@H]1C(C)C
Cadinol <α->	[C@@]1(C)(O)[C@@]2([H])[C@@]([H])(C=C(C)CC2)[C@H](C(C)C)CC1
Valerianol	C1[C@@]2(C)C(=CCC[C@@H]2C)CCC1[C@@](C)(C)O
Calamenen-10-ol <trans->	[C@]1(C)(O)C2C=CC(C)=CC=2C([C@H](C)C)CC1
Cadalene	C(C1C=CC(C)=C2C=CC(C)=CC=12)(C)C

Table S5. Docking solutions for each compound docked to *eeAChE*

Compound	Pose	PLP	Z_PLP	GS	Z_GS	CS	Z_CS	ASP	Z_ASP	AggScore
Tricyclene	Pose 1	48.94	1.82	15.21	0.34	24.78	0.47	24.22	0.79	0.85
Thujene < α->	Pose 1	52.56	2.56	25.03	1.39	23.45	1.16	24.42	1.8	1.73
	Pose 2	50.63	2.13	22.35	0.82	24.06	1.56	24.93	2.05	1.64
Pinene <α->	Pose 1	47.29	1.75	25.24	1	26.65	1.33	23.7	1.29	1.34
	Pose 2	45.6	1.32	25.53	1.06	26.04	0.95	22.84	0.98	1.08
Camphene	Pose 1	47.54	2.03	28.32	1.68	24.91	0.09	21.96	0.94	1.19
	Pose 2	44.58	1.3	21.18	0.43	26.19	1.17	22.97	1.24	1.03
Sabinene	Pose 1	48.47	1.86	23.43	1.09	24.07	1.76	22.64	1.39	1.52
	Pose 2	47.88	1.71	23.21	1.05	23.39	1.26	22.21	1.2	1.3
	Pose 3	44.96	0.95	30.52	2.38	22.21	0.39	21.61	0.93	1.16
Pinene <β->	Pose 1	44.07	1.25	28.26	2.35	26.5	1.31	23.14	1.42	1.58
	Pose 2	44.28	1.31	16.25	0.18	26.71	1.46	22.53	1.19	1.04
Myrcene	Pose 1	55.6	3.36	27.17	1.25	26.08	2.51	27.3	2.49	2.4
	Pose 2	47	0.97	35.57	3.29	23.15	0.71	21.95	0.84	1.45
	Pose 3	51.64	2.26	21.82	-0.06	25.13	1.93	22.95	1.15	1.32
Cymene <ϱ->	Pose 1	47.04	1.33	30.94	1.84	23.25	1	24.53	1.27	1.36
	Pose 2	50.61	2.23	29.55	1.58	22.01	-0.01	24.12	1.07	1.22
	Pose 3	42.65	0.23	23.84	0.53	24.69	2.17	25.4	1.69	1.15
	Pose 4	47.96	1.56	17.59	-0.63	24.45	1.97	25.04	1.52	1.11
	Pose 5	49.9	2.05	32.45	2.12	21.81	-0.17	22.68	0.38	1.09
	Pose 6	44.73	0.75	30.74	1.8	22.29	0.22	25.03	1.51	1.07
	Pose 7	46.59	1.22	26.63	1.04	22.9	0.72	24.38	1.2	1.04

Limonene	Pose 1	45.79	1.34	23.92	0.82	23.04	0.57	23.92	1.87	1.15
	Pose 2	44.1	0.85	24.76	0.99	23.78	1.2	23.18	1.43	1.12
	Pose 3	49.3	2.37	30	2.09	21.61	-0.64	21.52	0.44	1.07
	Pose 4	49.4	2.4	27.59	1.58	22.05	-0.27	21.54	0.46	1.04
Phellandrene <β->	Pose 1	49.85	2.22	24.23	1.31	25.2	1.84	24.53	1.68	1.76
	Pose 2	50.27	2.33	24.97	1.45	24.06	1.15	24.66	1.74	1.67
	Pose 3	45.73	1.17	32.47	2.82	23.84	1.01	21.33	0.28	1.32
Terpinene <y>	Pose 1	50.14	2.92	31.54	2.21	21.94	0	22.97	0.97	1.52
	Pose 2	42.79	0.56	23.68	0.7	24.12	1.83	24.51	1.67	1.19
	Pose 3	43.88	0.91	23.05	0.58	23.22	1.08	24.14	1.5	1.01
Chrysanthenol <cis->	Pose 1	45.86	1.11	24.87	1.02	23.34	0.4	25.05	1.1	0.91
Borneol	Pose 1	45.79	0.9	25.53	1.19	25.13	0.68	27.1	1.31	1.02
(2E,4E)-nona-2,4-dien-1-ol	Pose 1	55.65	2.87	27.05	1.17	25.08	1.66	27.07	2	1.92
	Pose 2	50.21	1.36	37.51	2.78	23.95	1.14	24.17	1.11	1.59
	Pose 3	49.37	1.12	20.41	0.15	23.84	1.09	26.81	1.92	1.07
Safranal	Pose 1	47.43	2	28.27	1.43	22.98	0.33	25.63	1.52	1.32
Chrysanthenyl acetate <trans->	Pose 1	51.82	1.61	34.86	1.61	20.62	0.21	31.02	1.13	1.14
	Pose 2	52.07	1.65	29.97	1.2	22.56	0.49	30.05	0.99	1.08
Bornyl acetate	Pose 1	57.91	2.24	27.63	1.53	26.43	1.1	28.86	1.17	1.51
	Pose 2	49.55	1.25	29.65	1.71	21.87	0.43	27.62	1	1.1
Pinocarvyl acetate <trans->	Pose 1	54.58	1.85	29.01	1.54	22.22	0.58	23.84	0.51	1.12
	Pose 2	48.85	1.18	25.22	1.17	22.84	0.66	28.51	1.08	1.02
Presilphiperfol-7-ene	Pose 1	58.5	2.29	29.72	0.49	32.88	0.84	29.57	1.08	1.18
Ylangene <α->	Pose 1	60.08	1.7	31.31	1.09	31.55	0.97	32.39	1.28	1.26
Bourbonene <β->	Pose 1	51.02	0.92	32.29	1.05	28.62	0.51	30.72	1.02	0.88
Linalool isobutanoate	Pose 1	59.94	1.28	27.73	0.65	24.36	0.72	28.69	0.64	0.82

Italicene <iso->	Pose 1	50.52	1.02	30.25	0.88	33.45	0.85	27.94	0.81	0.89
Longifolene	Pose 1	57.05	1.82	22.43	0.9	32.15	0.97	27.97	1.29	1.24
	Pose 2	48.6	1.07	22.79	0.92	32.87	1.03	27.43	1.24	1.07
Aromadendrene	Pose 1	49.35	0.67	29.49	1.36	34.57	2.28	33.72	2.27	1.65
	Pose 2	53.14	1.69	27.68	0.91	33.02	1.45	27.66	0.51	1.14
	Pose 3	48.91	0.55	30.51	1.62	32.33	1.08	29.85	1.15	1.1
	Pose 4	50.95	1.1	32.25	2.06	30.27	-0.03	29.53	1.05	1.05
Guaiadiene <6_9->	Pose 1	64.89	3.18	28.08	1.17	31.81	1.97	32.25	2.13	2.11
	Pose 2	57.93	1.82	30	1.56	31.17	1.66	28.91	1	1.51
	Pose 3	55.74	1.39	27.07	0.96	28.84	0.54	30.13	1.41	1.08
Spirolepechinene	Pose 1	58.99	1.55	30.21	1.12	30.85	0.91	30.15	1.17	1.19
	Pose 2	55.04	1.2	30.77	1.15	33.03	1.11	30.38	1.18	1.16
Humulene <α->	Pose 1	50.18	0.58	28.43	0.34	33.4	2.05	32.73	2.76	1.43
Muurolene <y>	Pose 1	59.97	1.7	23.05	1.48	33.11	1.15	31.04	1.01	1.33
Muurola-4(14)_5-diene <trans->	Pose 1	48.78	0.41	28.45	0.95	31.63	0.84	31.56	1.15	0.84
Calamenene <10_11-epoxy->	Pose 1	57.91	1.82	39.84	1.82	30.5	0.38	28.36	0.73	1.19
	Pose 2	48.28	0.28	33.69	1.14	35.92	1.37	32.51	1.4	1.05
Bicyclogermacrene	Pose 1	59.58	1.9	38.82	1.78	31.41	0.46	28.73	0.76	1.23
	Pose 2	61.94	2.2	30.11	0.77	34.18	0.93	29.75	0.92	1.21
	Pose 3	54.31	1.22	30.84	0.86	33.17	0.76	32.26	1.33	1.04
Farnesene <(E_E)-α->	Pose 1	65.93	1.54	31.33	0.69	34.33	1.34	32.28	0.94	1.13
	Pose 2	67.42	1.97	30.89	0.61	34.29	1.32	30.81	0.34	1.06
Cadinene <γ->	Pose 1	59.03	2.74	17.31	1.19	31.18	1.89	30.72	1.46	1.82
	Pose 2	54.57	1.3	9.48	0.11	32.24	2.5	33.2	2.28	1.55
	Pose 3	50.34	-0.06	17.7	1.24	31.53	2.09	30.95	1.53	1.2

Calacorene <α->	Pose 1	60.91	1.84	25.81	1.55	31.4	1.56	33.14	1.89	1.71
	Pose 2	62.7	2.3	16.05	0.03	31.38	1.55	32.86	1.79	1.42
	Pose 3	56.39	0.67	26.07	1.59	30.41	1.05	31.39	1.26	1.14
Spathulenol	Pose 1	60.06	1.82	30.72	1.35	34.79	1.23	34.5	1.29	1.42
Germacrene B	Pose 1	50.22	0.92	30.52	0.78	32.75	1.58	32.11	2.22	1.37
	Pose 2	51.99	1.48	28.56	0.47	33.53	2.01	28.76	1.14	1.28
	Pose 3	47.57	0.09	34.25	1.36	32.43	1.4	31.16	1.92	1.19
	Pose 4	51.39	1.29	31.15	0.87	32.69	1.54	28.41	1.03	1.18
Calacorene <β->	Pose 1	61.62	2.67	27.6	1.47	31.4	1.85	31.9	2.11	2.02
	Pose 2	60.29	2.3	24.72	1.23	27.78	-0.58	31.53	1.93	1.22
Atlantol <β->	Pose 1	65.89	1.71	29.15	1.16	33.93	0.85	34.92	0.77	1.12
	Pose 2	64.18	1.4	24.99	0.87	35.62	1.12	35.47	0.85	1.06
Caryophyllene oxide	Pose 1	61.98	2.29	27.49	0.34	34.84	1.03	34.07	1.21	1.22
Acorenol <α->	Pose 1	54.35	1.31	-4.89	1.55	24.82	0.53	26.19	0.9	1.07
Aromadendrene epoxide <allo->	Pose 1	58.95	2.11	44.67	2.07	36.49	1.17	30.46	0.68	1.51
Muurolol <α->	Pose 1	56.31	1.22	11.16	0.49	33	1.02	33.98	1.21	0.98
Cadinol <α->	Pose 1	55.14	1.13	15.85	1.22	30.82	0.86	29.82	0.74	0.99
Valerianol	Pose 1	54.6	1.01	19.91	1.01	30.68	0.7	31.85	1.01	0.93
Calamenen-10-ol <trans->	Pose 1	58.68	1.77	20.71	1.3	35.34	1.88	37.56	1.99	1.74
Cadalene	Pose 1	65.36	2.54	20.53	0.15	33.51	2.22	35.78	2.22	1.78
	Pose 2	59.33	1.03	21.47	0.32	34.35	2.66	36.39	2.44	1.61
	Pose 3	60.05	1.21	32.39	2.28	29.07	-0.12	32.43	1.04	1.1
	Pose 4	61.94	1.68	28.15	1.52	29.44	0.08	31.89	0.85	1.03

Table S6. ABFE Results for all complexes

Compound	Pose	ΔG (kcal/mol)	SEM (kcal/mol)
Tricyclene	Pose 1	-8.11	2.97
Thujene < α->	Pose 1	-11.12	1.51
	Pose 2	-13.53	0.67
Pinene <α->	Pose 1	-12.53	1.27
	Pose 2	-12.73	0.91
Camphene	Pose 1	-1.73	1.11
	Pose 2	-8.43	0.74
Sabinene	Pose 1	-5.94	1.18
	Pose 2	-6.1	1.61
	Pose 3	-13.82	1.41
Pinene <β->	Pose 1	-16.33	0.91
	Pose 2	-1.96	1.1
Myrcene	Pose 1	-13.27	3.26
	Pose 2	-10.86	1.99
	Pose 3	-10.34	1.42
Cymene <ϱ->	Pose 1	-11.03	1.25
	Pose 2	-6.89	1.52
	Pose 3	-4.38	1.29
	Pose 4	-11.24	1.44
	Pose 5	-6.99	1.38
	Pose 6	-6.04	1.93
	Pose 7	-8.64	2

Limonene	Pose 1	-5.88	1.35
	Pose 2	-8.92	0.88
	Pose 3	-7.15	1.31
	Pose 4	-8.99	1.2
Phellandrene <β->	Pose 1	-8.24	1.56
	Pose 2	-11.24	1.46
	Pose 3	-16.59	0.87
Terpinene <y>	Pose 1	-6.98	1.44
	Pose 2	-2.73	1.6
	Pose 3	-6.29	1.98
Chrysanthenol <cis->	Pose 1	-6.36	1.51
Borneol	Pose 1	-12.87	1.31
(2E,4E)-nona-2,4-dien-1-ol	Pose 1	-15.29	1.91
	Pose 2	-11.14	1.38
	Pose 3	-12.05	1.63
Safranal	Pose 1	-8.42	1.27
Chrysanthenyl acetate <trans->	Pose 1	-9.03	1.16
	Pose 2	-9.07	1.1
Bornyl acetate	Pose 1	-11.18	1.17
	Pose 2	-9.02	1.55
Pinocarvyl acetate <trans->	Pose 1	-12.79	1.48
	Pose 2	-4.76	1.44
Presilphiperfol-7-ene	Pose 1	-9.73	1.47
Ylangene <α->	Pose 1	-12.26	1.35
Bourbonene <β->	Pose 1	-4.14	1.8
Linalool isobutanoate	Pose 1	-7.23	1.91
Italicene <iso->	Pose 1	-12.15	1.86
Longifolene	Pose 1	-9.46	1.65

	Pose 2	-5.19	1.12
Aromadendrene	Pose 1	-11.58	2.29
	Pose 2	-6.18	1.93
	Pose 3	-0.9	1.45
	Pose 4	-7.01	1.58
Guaiadiene <6_9->	Pose 1	-10.34	1.6
	Pose 2	-4.7	1.74
	Pose 3	-7.46	1.86
Spirolepechinene	Pose 1	-20.22	0.93
	Pose 2	-12.59	1.46
Humulene <α->	Pose 1	-4.43	1.16
Muurolene <y>	Pose 1	-9.2	1.49
Muurola-4(14)_5-diene <trans->	Pose 1	-5.69	1.45
Calamenene <10_11-epoxy->	Pose 1	-10.04	1.51
	Pose 2	-9.84	1.41
Bicyclogermacrene	Pose 1	-9.04	2.08
	Pose 2	-11.62	1.47
	Pose 3	-6.91	2.13
Farnesene <(E_E)- α->	Pose 1	-11.12	2
	Pose 2	-12.31	2.4
Cadinene <γ->	Pose 1	-9.96	0.83
	Pose 2	-14.93	1.66
	Pose 3	-6.82	2.02
Calacorene <α->	Pose 1	-9.35	1.07
	Pose 2	-3.88	1.2
	Pose 3	-8.02	1.64
Spathulenol	Pose 1	-3.96	1.46

Germacrene B	Pose 1	-13.28	1.9
	Pose 2	-7.22	1.45
	Pose 3	-7.96	0.82
	Pose 4	-4.01	2.26
Calacorene <β->	Pose 1	-1.75	1.32
	Pose 2	-8.95	1.71
Atlantol <β->	Pose 1	-11.8	2
	Pose 2	-9.26	1.68
Caryophyllene oxide	Pose 1	-4.69	1.81
Acorenol <α->	Pose 1	-12.6	1.25
Aromadendrene epoxide <allo->	Pose 1	-15.81	2.53
Muurolol <α->	Pose 1	-4.05	3.7
Cadinol <α->	Pose 1	-7.89	1.88
Valerianol	Pose 1	-1.08	1.28
Calamenen-10-ol <trans->	Pose 1	-12.92	1.18
Cadalene	Pose 1	-11.79	1.74
	Pose 2	-9.86	1.76
	Pose 3	-13.02	3.31
	Pose 4	-0.26	1.89

Values are reported as ΔG and the associated block-based uncertainty estimate, labeled SEM in the BAT.py output, in kcal/mol. These uncertainties refer to individual calculations and do not include variability between starting poses or systematic model errors. Pose numbering corresponds to Supplementary Table S3

Figure S3. Structural comparison of the donepezil-binding region in eeAChE and hsAChE. (a) Surface representation of the eeAChE model showing the transferred donepezil pose (magenta) and an enlarged view of the aligned residues (eeAChE, green; hsAChE, yellow). Residue labels follow eeAChE numbering. (b) Correspondence of the 32 cavity residues and their prediction confidence scores (pLDDT) in eeAChE. Nonidentical residue pairs are highlighted in gray and bold.

(a)

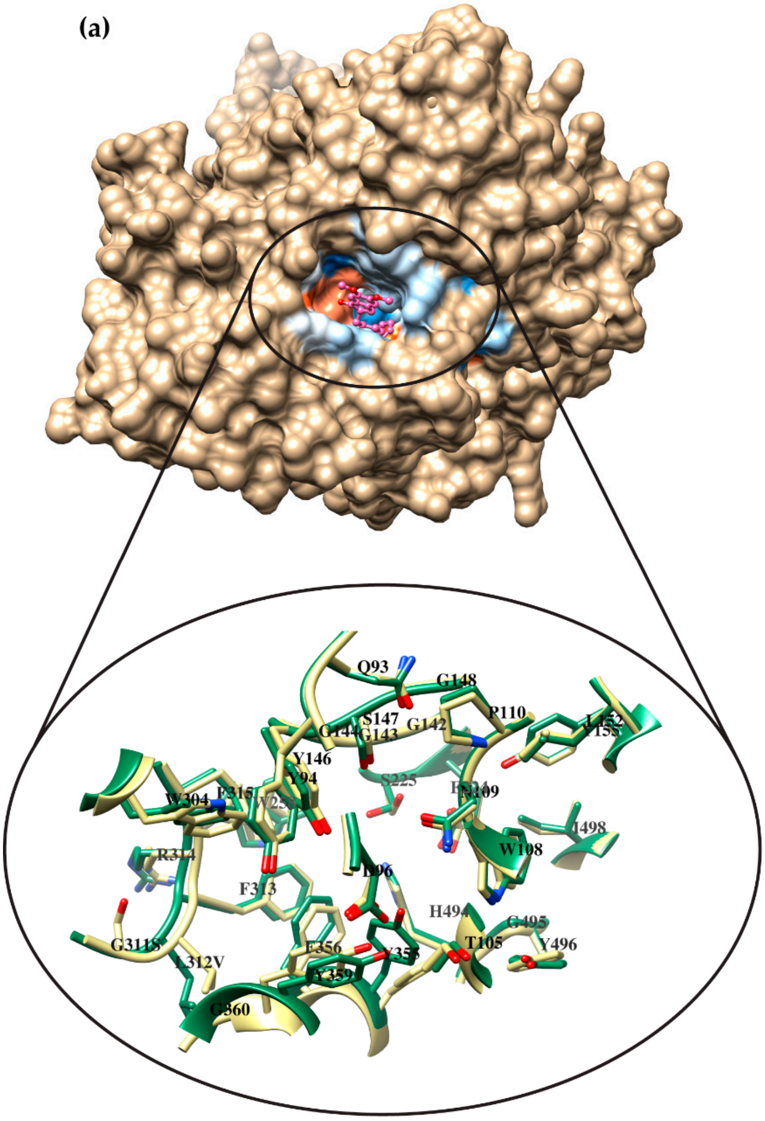

(b)

eeAChE<br>E. electricus	hsAChE<br>H. sapiens	pLDDT<br>eeAChE
Q93	Q71	98.69
Y94	Y72	97.81
D96	D74	96.56
T105	T83	96.44
W108	W86	98.44
N109	N87	98.50
P110	P88	98.38
G142	G120	98.75
G143	G121	98.38
G144	G122	98.12
Y146	Y124	98.56
S147	S125	98.69
G148	G126	98.62
L152	L130	98.62
Y155	Y133	98.75
E224	E202	98.94
S225	S203	98.81
W258	W236	94.44
W304	W286	96.25
**G311**	**S293**	87.25
**L312**	**V294**	91.00
F313	F295	92.62
R314	R296	94.81
F315	F297	96.69
Y355	Y337	96.62
F356	F338	96.62
Y359	Y341	94.12
G360	G342	89.25
H494	H447	98.69
G495	G448	98.31
Y496	Y449	98.62
I498	I451	98.81
